# Meta‐Attention Deep Learning for Smart Development of Metasurface Sensors

**DOI:** 10.1002/advs.202405750

**Published:** 2024-09-09

**Authors:** Yuan Gao, Wei Chen, Fajun Li, Mingyong Zhuang, Yiming Yan, Jun Wang, Xiang Wang, Zhaogang Dong, Wei Ma, Jinfeng Zhu

**Affiliations:** ^1^ Institute of Electromagnetics and Acoustics and Key Laboratory of Electromagnetic Wave Science and Detection Technology Xiamen University Xiamen Fujian 361005 China; ^2^ State Key Laboratory of Physical Chemistry of Solid Surfaces Department of Chemistry College of Chemistry and Chemical Engineering Xiamen University Xiamen 361005 China; ^3^ Institute of Materials Research and Engineering (IMRE) Agency for Science, Technology and Research (A*STAR) 2 Fusionopolis Way, Innovis # 08‐03 Singapore 138634 Republic of Singapore; ^4^ Department of Materials Science and Engineering National University of Singapore 9 Engineering Drive 1 Singapore 117575 Singapore; ^5^ College of Information Science and Electronic Engineering Zhejiang University Hangzhou 310027 China

**Keywords:** bound states in the continuum, explainable deep learning, metasensors, metasurface, transformer

## Abstract

Optical metasurfaces with pronounced spectral characteristics are promising for sensor applications. Currently, deep learning (DL) offers a rapid manner to design various metasurfaces. However, conventional DL models are usually assumed as black boxes, which is difficult to explain how a DL model learns physical features, and they usually predict optical responses of metasurfaces in a fuzzy way. This makes them incapable of capturing critical spectral features precisely, such as high quality (Q) resonances, and hinders their use in designing metasurface sensors. Here, a transformer‐based explainable DL model named Metaformer for the high‐intelligence design, which adopts a spectrum‐splitting scheme to elevate 99% prediction accuracy through reducing 99% training parameters, is established. Based on the Metaformer, all‐dielectric metasurfaces based on quasi‐bound states in the continuum (Q‐BIC) for high‐performance metasensing are designed, and fabrication experiments are guided potently. The explainable learning relies on spectral position encoding and multi‐head attention of meta‐optics features, which overwhelms traditional black‐box models dramatically. The meta‐attention mechanism provides deep physics insights on metasurface sensors, and will inspire more powerful DL design applications on other optical devices.

## Graphical abstract

This study develops an explainable deep learning architecture named as Metaformer to facilitate intelligent design of metasurface sensors and explains the learning mechanism by revealing a series of component stages for different attentions of multiple heads. The Metaformer model provides deep physics insights on metasurface sensors and will inspire more powerful deep learning design applications on other optical devices.

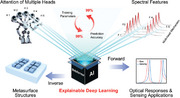

## 1 Introduction

Optical metasurfaces are artificial electromagnetic materials consisting of meta‐atoms within sub‐wavelength dimensions. They demonstrate extraordinary light‐matter interactions and have the potential for many optical applications.^[^ ^1^, ^2^, ^3^ ^]^ Particularly, they are widely investigated on nondestructive label‐free sensing for chemical and biomedical detection, since they could have near‐field concentration effects inspired by various mechanisms, such as plasmonics and quasi‐bound state in the continuum (Q‐BIC).^[^ ^4^, ^5^, ^6^, ^7^, ^8^, ^9^ ^]^ The optical sensors based on metasurfaces are also called as metasensors, which include two important categories for applications on molecular fingerprint detection and refractive index (RI) sensing, respectively.^[^ ^10^, ^11^ ^]^ On one hand, a molecular fingerprint metasensor is usually used to detect a trace sample's characteristic peaks of molecule vibration modes through infrared (IR) absorption spectroscopy. Such a metasensor is typically designed with multiple resonance wavelengths in the IR spectrum, which correspond to the featured absorption peaks of trace molecular fingerprint. This can overcome the significant dimension mismatch between the IR wavelengths (>3000 nm) and target molecules (<10 nm), enhance the localized near field surrounding the trace samples, and boost the fingerprint signal dramatically.^[^ ^12^ ^]^ On the other hand, an RI metasensor is used to detect the change of material refractive index due to the specific coatings of environmental gas, liquid, biomolecules, or chemical molecules, which facilitates their identification and quantification. It has two important physical parameters, including the bulk refractive index sensitivity (BRIS) and surface refractive index sensitivity (SRIS).^[^ ^13^, ^14^, ^15^ ^]^ During the design of RI metasensor, one needs to optimize the geometry of metastructure for the target sensitivity performance or to pursue a metastructure goal according to a customized sensitivity inversely.^[^ ^16^, ^17^ ^]^ In general, the design for both fingerprint metasensors and RI metasensors requires the redundant optical simulation, massive structure parameter scan, and dedicated optimization process, which is not only time‐consuming but also highly dependent on experiences of metasurface engineering. Nowadays, a powerful design method is quite in demand for developing these two metasensors.^[^ ^18^ ^]^

Recently, along with its bloom in science and technologies,^[^ ^19^, ^20^, ^21^ ^]^ deep learning (DL) has become a state‐of‐the‐art approach for metasurface design.^[^ ^22^, ^23^, ^24^, ^25^ ^]^ Many kinds of artificial neural networks (NNs), such as multilayer perceptron (MLP), convolutional neural networks, and generative adversarial networks, have been employed as the black‐box models for the forward and inverse design from meta‐atoms to optical responses.^[^ ^26^, ^27^, ^28^, ^29^, ^30^, ^31^, ^32^, ^33^, ^34^, ^35^, ^36^, ^37^ ^]^ These DL techniques provide opportunities to search structure parameter spaces in a more efficient way, leading to the data‐driven, on‐demand design of metasurfaces and related metadevices.^[^ ^38^ ^]^ However, there is still a lack of DL paradigms for the universal development of metasurface‐based sensors, especially for the smart design of fingerprint metasensors and RI metasensors. This is because conventional DL models are assumed as black boxes, which usually predict optical responses of metasurfaces in a fuzzy way. This makes them incapable of capturing high quality (Q) spectral peaks or valleys precisely, which influences the sensing performance of metasurfaces dramatically. The failure in comprehending critical spectral features hinders the effective use of these DL frameworks in metasensor design. Currently, it is quite essential to break the black‐box limitation and build an explainable DL architecture to notice specific spectral features for powerful metasensor design.

**Figure 1:**
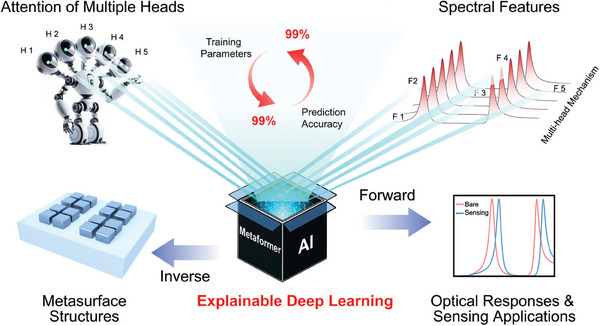
Conceptual diagram of explainable deep learning model named Metaformer for smart development of metasurface sensors.

Here, we develop an explainable DL architecture named as Metaformer to facilitate intelligent design of metasensors with high Q factors as shown in **Figure** **1**. The inverse design is used to predict the metasurface structures according to the input of spectral responses in various environments. The forward design is adopted to obtain the spectral responses for different environments to obtain the target sensitivity. The Metaformer is based on the transformer framework and has a unique mechanism of multi‐head self‐attention, which enables the high‐efficiency capture of different spectral feature details. It demonstrates a significant prediction error reduction of 99% with the 99% decrease of training parameters than conventional transformer model. The transformer model has shown the potential for designing metamaterial absorbers, while it has not been applied on the development of metasensors with more complex environmental changes. Moreover, the underlying learning mechanisms of the transformer model have yet to be clearly explored and elucidated.^[^ ^39^ ^]^ The multi‐head attention is critical for the Metaformer model, which is widely adopted to interpret the model feature extraction mechanism and can assign different weights to various input subsequences based on their relevance for producing accurate predictions.^[^ ^40^, ^41^ ^]^ Here, we explain the learning mechanism by revealing a series of component stages for different attentions of multiple heads within the network. We find that different heads can focus on various spectral regions with specific curve features. Based on the model, we develop a tool for the quick flexible design of metasensors. We design and fabricate the Q‐BIC‐based metasensors as proof‐of‐concept examples, and measure their optical responses for fingerprint and RI sensing, respectively. The good agreement between the theory and experiments implies the great potential of Metaformer on powerful intelligent design of metasensors and other metadevices.

## 2 Results and Discussion

### 2.1 The Metaformer Model and Physical Paradigm Based on Q‐BIC

**Figure 2:**
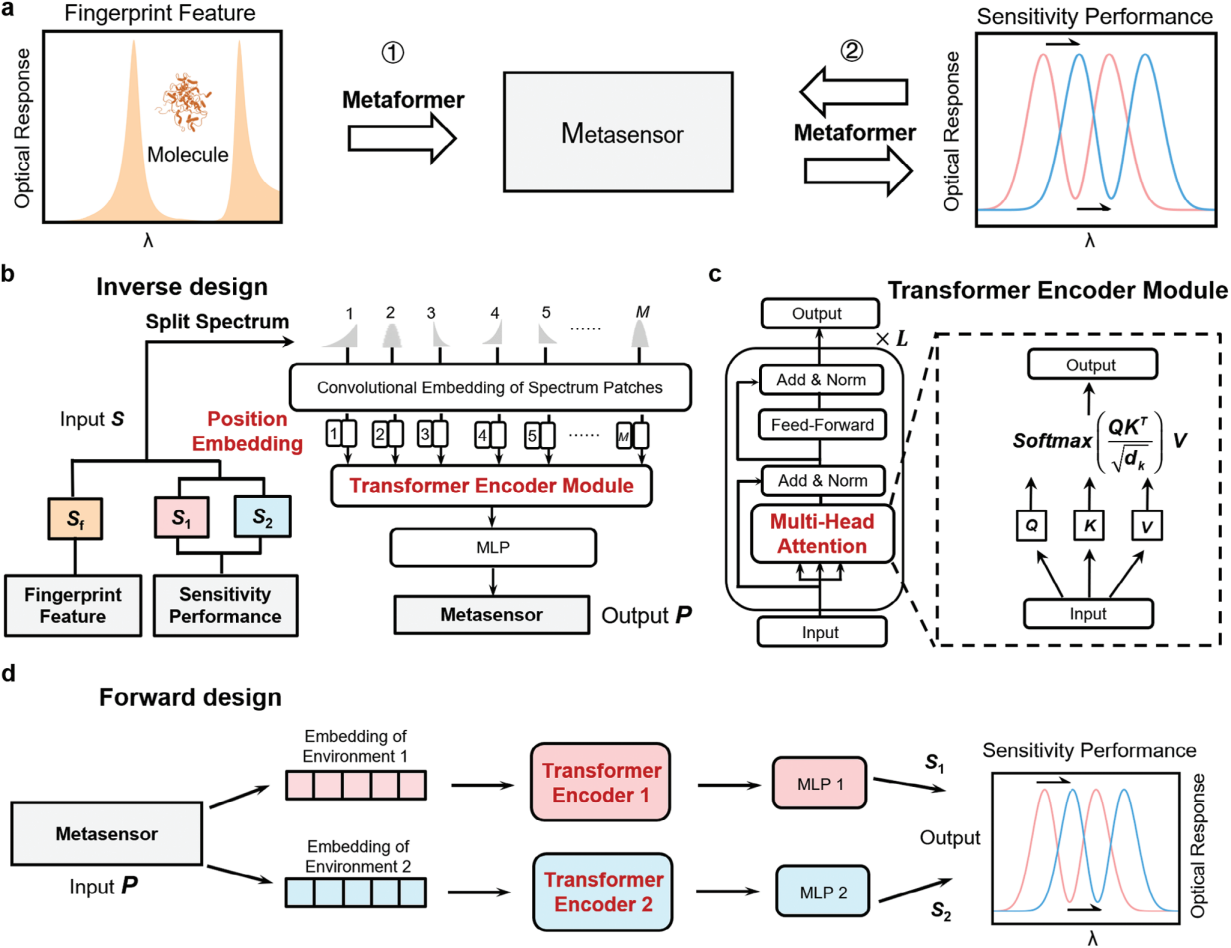
a) Design functions of Metaformer for fingerprint and RI metasensors. b) Inverse design architecture of metasensor for on‐demand fingerprint feature and sensitivity performance. c) Network architecture of transformer encoder module. d) Forward design architecture for predicting sensitivity performance of metasensor.

The Metaformer architecture is illustrated in **Figure** **2**. It contains two design functions of metasensors shown in Figure 2a. One is the inverse design of on‐demand metastructure according to a spectrum of molecule fingerprint with featured peaks, and the other is the forward and inverse design from the metastructure to optical sensitivity. In the inverse design process for fingerprint and RI metasensing, the Metaformer takes the optical spectrum vector ***S*** as the input and the output is the vector ***P*** of metastructure parameters, as denoted in Figure 2b. Particularly, the design of a fingerprint metasensor needs the spectrum input of ***S*_f_**, while the design of an RI metasensor requires the spectrum inputs of ***S***_1_ and ***S***_2_ for two different environments, which are originated from the definition of sensitivity performance, including the BRIS and SRIS. The BRIS is defined as ***S***_BRIS_ = (λ_2_ − λ_1_)/(*n*_2_ − *n*_1_), where λ_1_ and λ_2_ are the resonance wavelengths of two different environments, respectively; *n*_1_ and *n*_2_ denote the corresponding values of enviromental refractive index.^[^ ^42^ ^]^ The SRIS can be briefly described by ***S***_SRIS_ = (λ_2_ − λ_1_)/(*nt*), where *n* and *t* represent the refractive index and conformal thickness of trace analyte coated on the metasensor, respectively.^[^ ^43^ ^]^ The input spectrum is split into *M* patches to overcome the significant dimension mismatch between the input ***S*** and output ***P***. Each split spectrum patch is embedded by a convolution operation, which is followed by a set of positional encoding. This process generates a sequence of vectors, and it is fed to the transformer encoder module. This module is linked to an MLP layer, and leads to the output ***P*** for the predicted metastructure parameters. The transformer encoder module consists of *L* identical layers, as illustrated in Figure 2c. Each layer consists of a multi‐head attention component followed by a feed‐forward MLP. The configuration of residual connection and layer normalization are adopted for both the attention and MLP components.^[^ ^44^ ^]^ In each head of attention, the input sequence is multiplied with three learnable weight vectors, and converted to the vector including the sub‐vectors of query, key and value (*Q, K, V*). The self‐attention mechanism is adopted and described by the following equation,^[^ ^45^ ^]^

(1) $$\mathrm{Attention}\;\left(Q,K,V\right)=\;\mathrm{Softmax}\left(\frac{QK^{T}}{\sqrt{d_{k}}}\right)V$$

where *d_k_* represents the dimension of *Q* and *K*. For each pair of *Q* and *K*, the dot products of query with all the keys are calculated and divided by $\;\sqrt{d_{k}}$. These results are applied on the Softmax function to get the attention weights on the values. The attentions from all heads are concatenated together to obtain the result of multi‐head attention mechanism as the following equation,

(2) $$\begin{array}{ccc} & & \mathrm{MultiHead}\;\left(Q,K,V\right)\\ & & =\;\mathrm{Concat}\;\left(\mathrm{Hea}d_{1},\text{…}\mathrm{Hea}d_{i},\text{…},\;\mathrm{Hea}d_{Z}\right)W^{O} \end{array}$$

where *Z* represents the number of head, Head*_i_* is the attention result of the *i* ^th^ head, and *W* ^0^ denotes the weight parameter matrix related to all the heads. The result of multi‐head attention is fed into the MLP layer, as shown in Figure 2b. The forward design process from the metasensor to sensitivity performance is illuminated in Figure 2d, where the input ***P*** is embedded by two sets of data associated with different sensing environments, respectively. This operation enables the input vector from low dimension to high dimension. The two vectors after embedding are fed to two separate transformer encoder modules, whose network architectures are the same as Figure 2c. The two transformed sequences go into their corresponding MLP layers, and generate the spectrum vectors ***S***_1_ and ***S***_2_ for two different environments, respectively, which are used to calculate the sensitivity of metasensor.

**Figure 3:**
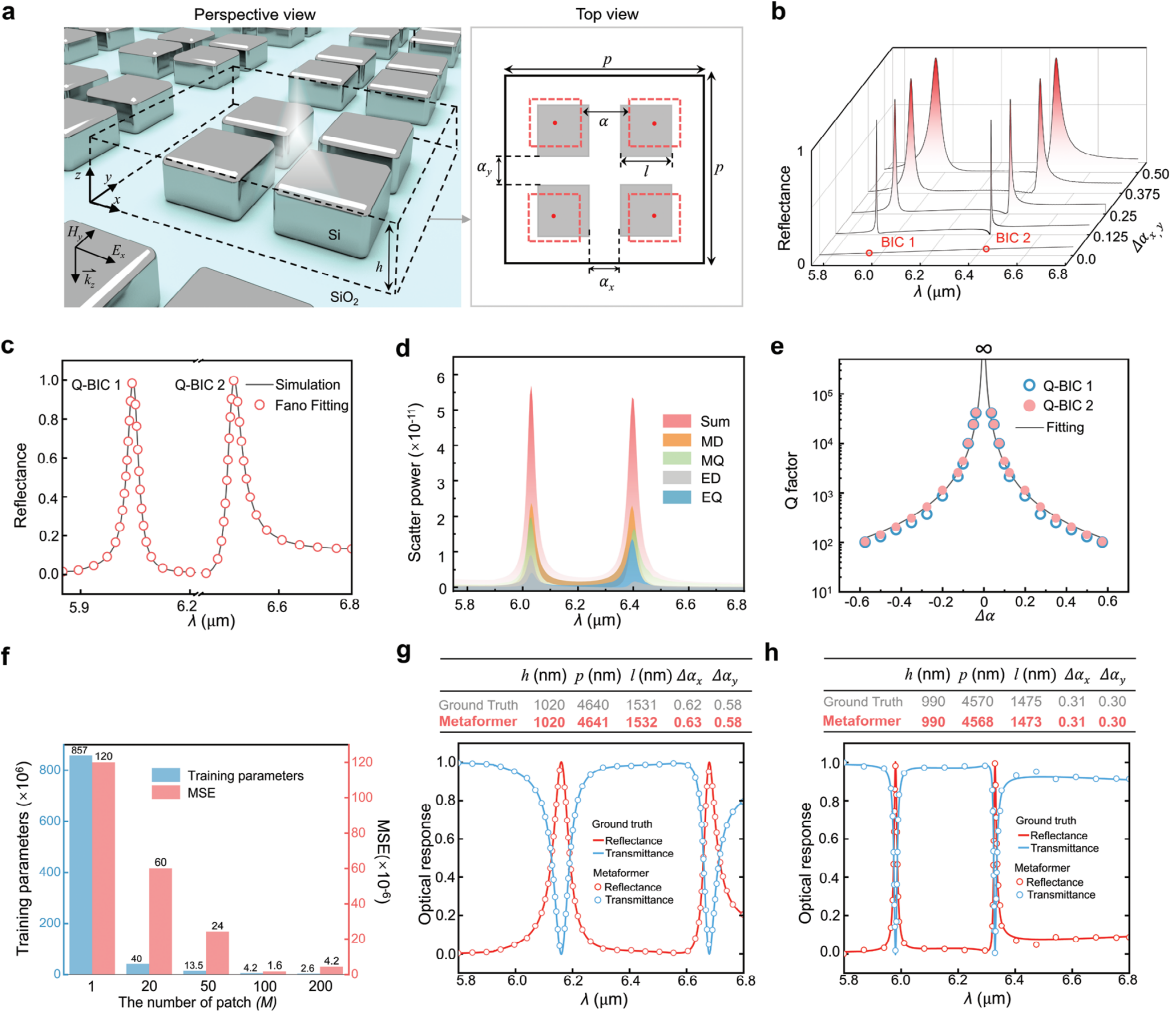
a) Schematic drawing of Q‐BIC‐based all‐dielectric metasensor, where the inset shows the top view of a single meta‐atom. b) The evolution of reflection curves with changed Δ*α*. c) Simulated spectrum of reflectance and Fano fitting for Q‐BIC 1 and Q‐BIC 2. d) Scatter power as a function of wavelength by multipole decomposition. e) Q factor functions of Δ*α* for two Q‐BIC resonances. Training parameter number and MSE f) for a series of different patch values. g,h) Two randomly selected instances of inverse and forward design from testing sample space.

In order to evaluate the design functions of Metaformer, we apply it to the physical model of all‐dielectric metasensors based on Q‐BIC.^[^ ^46^ ^]^ Such metasensors have lower optical damping than plasmonic metasensors^[^ ^47^ ^]^ and could also support highly confined optical mode with notable high‐Q resonance features for enhancing both fingerprint and RI sensing.^[^ ^48^ ^]^ As shown in **Figure** **3**a, we adopt the paradigm of symmetry‐broken metasurface, whose meta‐atom consists of a top patterned Si layer with four square patches and a fused silica substrate. The optical parameters of Si and fused silica are obtained from the reference.^[^ ^49^ ^]^ The geometry of meta‐atom is determined by the period (*p_x_* = *p_y_* = *p*), length (*l*) of patch side, height (*h*) of patch, and gaps between two adjacent patches along *x*‐axis (*α_x_*) and *y*‐axis (*α_y_*) directions. Originally, when the four meta‐atom patches are located at the centers of four quadrants in a 2D period, respectively, we denote the spacing between two adjacent patches as *α*, which represents the in‐plane symmetry‐protected geometry. When the patches deviate from the centers of four quadrants, the values of *α_x_* or *α_y_* are not equal to *α*, which breaks the in‐plane symmetry of the four patches.

Here, we define the asymmetric factors of geometry as Δ α_*x*_ = (α − α_*x*_ )/α and Δ α_*y*_ = (α − α_*y*_ )/α. When Δ*α_x_* = Δ*α_y_* = 0, the meta‐atom maintains the symmetry and there are two BIC modes (BIC 1 and BIC 2) without radiation leakage,^[^ ^50^ ^]^ as shown in Figure 3b. As Δ*α_x_* and Δ*α_y_* both increase to 0.125, the symmetry is broken and the electromagnetic energy is released through two resonance modes with ultra‐narrow line widths, namely “Q‐BIC 1” and “Q‐BIC 2”. The line widths of resonances are broadened along with the rise of the asymmetric factors. As observed in Figure 3c, the simulated resonance curves of Q‐BIC 1 and Q‐BIC 2 can be fitted by using Fano Formula and indicate good agreement with the asymmetric Fano line shapes.^[^ ^51^ ^]^ The two Q‐BIC resonances can be further analyzed by the multipole decomposition method (see more details in Supporting Information), as shown in Figure 3d. The metasensor supports the modes of electric dipole (*ED*), magnetic dipole (*MD*), electric quadrupole (*EQ*), and magnetic quadrupole (*MQ*), where the major components of these two Q‐BIC resonances are *MD* modes. In particular, the resonances of Q‐BIC 1 and Q‐BIC 2 demonstrate the inverse quadratic law *Q* = *Q*_0_(Δ*α*)^−2^, where *Q*_0_ is a fitting constant, *Q* = λ_0_ /FWHM denotes the quality factor of resonance, and FWHM represents the full width at half maximum,^[^ ^52^, ^53^ ^]^ as shown in Figure 3e. The theory and simulation results from Figure 3b–e indicate that the spectra of the proposed physical model typically have two high‐Q resonances, which can be used to verify whether the Metaformer model can notice the specific spectral features for developing Q‐BIC‐based metasensors effectively.

To assess the network performance of Metaformer, we define its loss function by calculating the mean square error (MSE) between the predicted results and the ground truth, which is described as below,^[^ ^39^ ^]^

(3) $$Loss\;=\frac{1}{N_{s}}\;\sum_{i\;=\;1}^{L}{\left(T_{i}-{\tilde{T}}_{i}\right)}^{2}$$

where *T_i_* denotes the spectrum or metastructure predicted by the Metaformer, ${\tilde{T}}_{i}$ is the simulated spectrum or metastructure, and *N_s_* is the number of spectral sample points. Conventional transformer model has been widely used in natural language processing and can deal with complex tasks with large datasets. Its memory and computation requirements grow quadratically with the sequence length, which makes it inadvisable to handle long sequences.^[^ ^54^ ^]^ In the model of inverse design, one needs a great number of spectral sample points with high resolution to characterize high‐Q resonances of Q‐BICs. On this condition, the use of conventional transformer network would lead to a dramatical increase of parameters and make it difficult to converge due to long spectral sequences.^[^ ^55^ ^]^ With the aim to overcome this problem, we combine the advantages of typical transformer with the inspiration from the patch operation in vision transformer for metasensor,^[^ ^56^ ^]^ we split each spectrum into *M* patches and investigate the influence of *M* value on the training parameters and MSE of testing samples in Figure 3f. As *M* increases, the dimensions of spectrum will become smaller when the whole spectrum is divided into different patches, which causes the number of training parameters has a significant reduction than the conventional transformer. The optimal patch number of *M* is 100, which leads to the lowest MSE of 1.6 × 10^−6^. Compared with the conventional transformer, such a result demonstrates an MSE reduction up to 99% by decreasing the training parameters of 99%. A typical NN can be used for spectral prediction of metastructures, but it is usually difficult to precisely predict sharp spectral responses for a simple NN with limited sample points.^[^ ^57^, ^58^, ^59^ ^]^ The spectrum input sequences contain rich physical information, while the resonance peaks with high‐Q features occupy a smaller proportion of the sample points than the other flat regions and have stronger variation around resonance wavelength, which makes the prediction much more challenging^[^ ^60^ ^]^ (See more details in Supporting Information). During the design of metasensors, it is crucial to accurately predict resonance wavelengths of a spectrum with high‐Q features. A NN is usually incapable to achieve the accuracy requirements. Therefore, we developed the Metaformer model with higher accuracy to achieve the smart design of metasensor. Compared to the classical MLP model, the result indicates an MSE decrease of 44.8% by reducing the training parameters of 54.8% (see Table S2, Supporting Information). This implies the better prediction performance of our network on inverse design than conventional approaches. Here, we randomly pick up two testing instances of the Metaformer learning model and compare the design results with the ground truth in Figure 3g,h. The relative errors for the two sets of predicted metastructure parameters in inverse design are less than 0.3%. These predicted metastructure parameters are further used to predict two corresponding spectra by forward design, respectively. Compared with the ground truth, they indicate the MSE values of spectrum as low as 4.0 × 10^−5^ and 1.7 × 10^−4^, respectively. Therefore, both the forward and inverse processes by the Metaformer demonstrate good consistency with the ground truth, which suggests its high‐accuracy design performance (see more details in Figure S4, Supporting Information). Particularly, our method can be widely applied to other optical devices with more complicated spectra features.

### 2.2 Explainable Learning Mechanism for the High Performance of Metaformer

**Figure 4:**
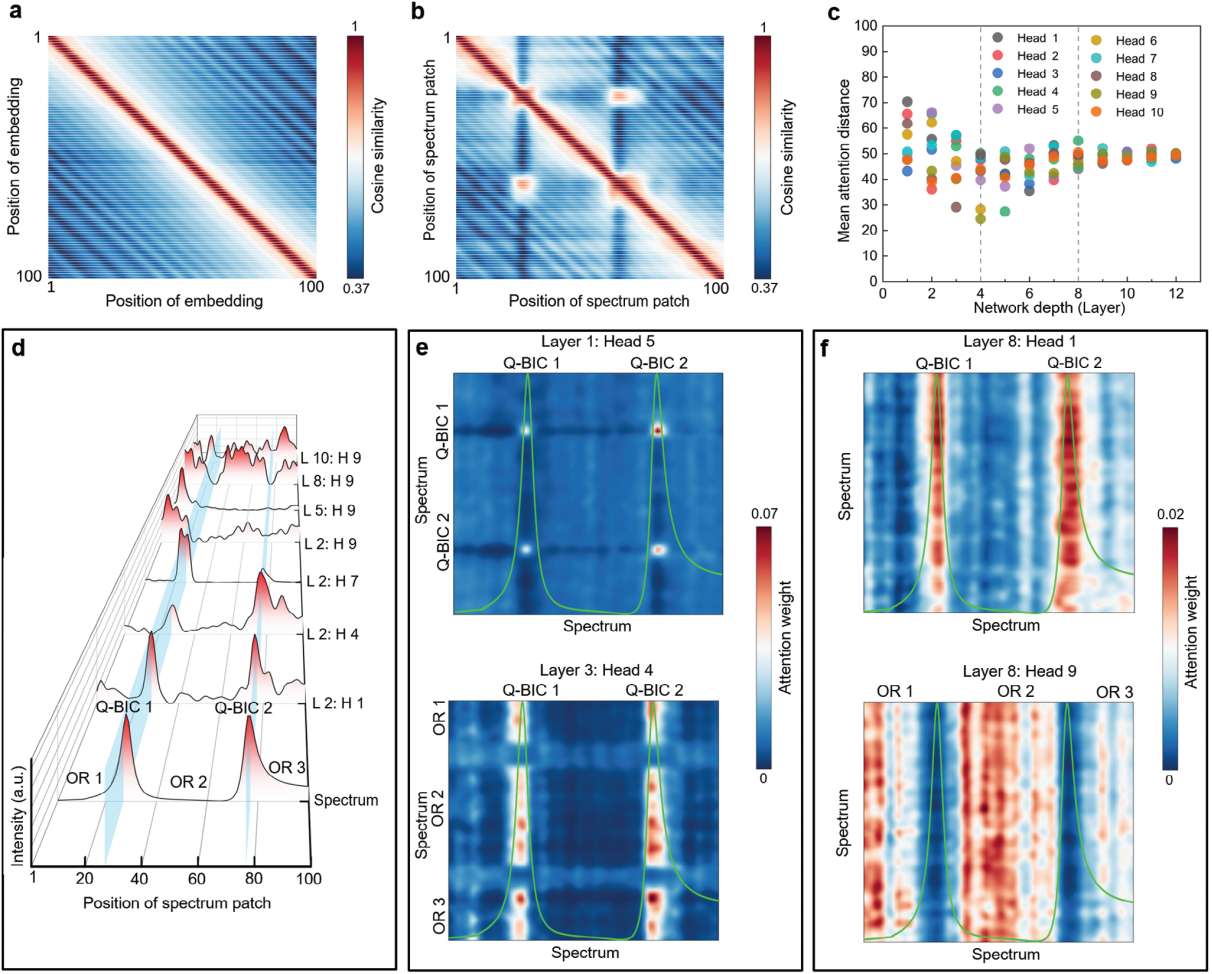
a,b) Cosine similarity heatmaps of position encoding matrix without and with spectrum patches, respectively. c) Mean attention distance as a function of network layer and head. d) Spectral intensity and attention weight intensities and as functions of patch position for various network layers and heads. Here, the symbols L and H denote the layer and head, respectively; the numbers behind them represent the corresponding network depth and head order, respectively. e,f) Heatmaps of attention weight for various network layers and multiple heads.

We interpret the high prediction accuracy of Metaformer especially by illuminating its learning mechanism for the inverse design. In the network of inverse design, the position encoding plays a critical role for learning. It ensures that the model acquires the position information of spectral sequence, which is calculated by the following equations,^[^ ^45^ ^]^

(4) $$\begin{array}{c} \;PE_{\left(pos,2i\right)}=\;sin\left(\frac{pos}{10000^{2i/d_{\mathrm{model}}}}\right)\\ \;PE_{\left(pos,2i+1\right)}=\;cos\left(\frac{pos}{10000^{2i/d_{\mathrm{model}}}}\right)\; \end{array}$$

where *pos* is the position of spectrum patch in the sequence, *d*_model_ = 128 denotes the embedding dimension, *i* is the index of dimension. In this process, all the spectrum patches are expanded to 128 dimensions, and the operation by Equation (4) embeds the patch position information (see more details in Figure S4, Supporting Information), which aims to make the input data ordered for the following processing. We plot the cosine similarity of position embedding in **Figure** **4**a, which reflects that the closer positions tend to have a higher level of similarity. For instance, each position has the highest cosine similarity of 100% with itself, as shown on the red diagonal band in Figure 4a. Thus, the position embedding can encode the distance of input sequence by the similarity calculation. When the sequenced patches of one optical response are combined with the position embedding, the plot of cosine similarity maintains the diagonal feature of position correlation, as observed in Figure 4b. Since the spectrum has two significant peaks of the typical Q‐BIC‐based resonances around the two patch positions of 26 and 63, respectively, the high similarities surrounding Patch (26, 26) and Patch (63, 63) are extended to a larger region. The levels of similarity around Patch (26, 63) and Patch (63, 26) are enhanced due to the similar spectral peak shapes of Q‐BIC resonances between Patch 26 and 63. The result of Figure 4b demonstrates that the position information is effectively embedded into the spectrum sequence.

We next investigate the effects of multi‐head attention mechanism for the learning of inverse design. Previous studies have reported that it allows the integration of global information in the transformer encoder.^[^ ^56^ ^]^ Here, we evaluate the degree of our network on utilizing this capability. As shown in Figure 4c, we adopt 12 transformer encoder layers with 10 heads and calculate the mean attention patch distance of the spectrum through the layers for each head based on the attention weights. The learning for the mean attention distance of a randomly selected spectrum can be divided into three stages according to the depth of network layers. There are some heads attending to most of the spectrum patches even in the lowest layers of Stage I. This implies that the Metaformer is indeed empowered by the function of integrating information globally. During Stage I, the maximum and minimum mean attention distances gradually decrease from a high position to a low one with the increase of network depth. After they approach the lowest positions, they continue to rise to the higher positions at Stage II. In the highest layers of Stage III, the mean attention distances for all heads converge to the position of around the 50th patch, which is exactly a half of the distance for all the 100 spectrum patches. The ultimate convergent position might be attributed to the typical features with two Q‐BIC resonance peaks for a random spectrum of metasensor, whose two resonances are driven by breaking the in‐plane symmetry along the directions of *x*‐axis (α*_x_*) and *y*‐axis (α*_y_*), respectively. These two directions are orthogonal and could make the two Q‐BIC resonances independent to each other. Therefore, regarding a randomly selected spectrum, the positions of two Q‐BIC resonance peaks could be located at both sides of the 50th patch equiprobably.

In order to reveal the learning capability of Metaformer, we plot the attention weight intensities as functions of patch position for a series of network layers and heads and compare them with the randomly selected spectrum for input, as shown in Figure 4d. Typically, there are five segments for the spectrum, denoting Q‐BIC 1 and Q‐BIC 2 resonance peaks and three off‐resonance (OR) regions. For the network depth of Layer 2, Head 1 exhibits two high intensity peaks of attention weight corresponding to the two Q‐BIC resonances; while Head 4, Head 7, and Head 9 show remarkable attention weight intensities on the spectrum patches for segments of Q‐BIC 2, Q‐BIC 1 resonances, and OR 1, respectively. This demonstrates that the multiple heads for a fixed layer (even the earlier layer) can notice the representative segment features for an entire spectrum. On the other hand, for the fixed head number of 9, Layers 2, 5, 8, and 10 indicate high attention weight intensity for the OR spectral segments; Layers 2 and 5 show high attention weights for the patches of OR 1, and the patches of OR 1, OR 2, and OR 3 tend to have high attention weights as the layer order numbers are increased to 8 and 10, respectively. The result in Figure 4d illuminates the good learning ability by multi‐head attention mechanism through a sequence of network layers.

Furthermore, we plot the learning heatmaps of multi‐head attention weights for some representative layers and heads of the Metaformer in Figure 4e,f. There are four bright spots with high attention weights for Head 5 of Layer 1 in Figure 4e, which indicates that the Metaformer notices and assigns more attention weights to the two significant peak features of Q‐BIC 1 and Q‐BIC 2 resonances. The four spots imply the self‐attention of each Q‐BIC resonance and the mutual attention to each other for the two Q‐BIC resonances. The heatmap of attention weight in Head 4 of Layer 3 shows six distinct high‐value regions. They imply the mutual attention between each Q‐BIC resonance peak and each OR segment of the spectrum. In Figure 4f, the heatmap of attention weight in Head 1 of Layer 8 exhibits two notable bands with high attention weights, which reflects the mutual attention between each Q‐BIC resonance peak and the entire spectrum; on the contrary, there are three bright bands of high attention weights in Head 9 of Layer 8, indicating the mutual attention between each OR spectral segment and the whole spectrum (see more details in Figure S6, Supporting Information). The results in Figure 4 reflect that different heads going through network layers attend to various spectral segments with specific curve features, which elucidates high prediction precision on spectral details and facilitates good performance on the smart design of Q‐BIC‐based metasensors.

### 2.3 Metasensor Design for IR Fingerprint Detection

**Figure 5:**
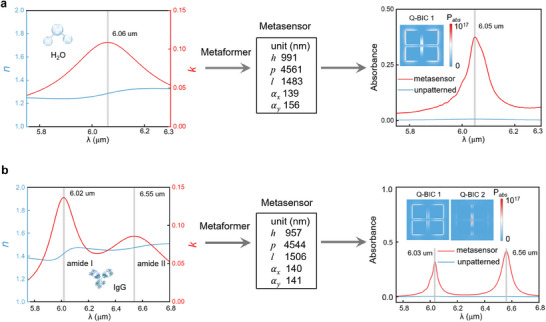
The IR complex refractive index, predicted metastructure parameters, fingerprint absorbance spectra of metasensor, and unpatterned substrate for (a) H_2_O and (b) IgG. The insets denote the absorbed power distributions at the fingerprint peaks induced by the corresponding Q‐BICs.

Based on the learning mechanism research of Metaformer, we continue to conduct the intelligent design of fingerprint metasensors based on Q‐BICs, which are aimed to detect the characteristic peaks on IR absorbance spectra of target molecules. We first test the fingerprint metasensor design for detecting a trace H_2_O sample layer of 50 nm thick. The IR complex refractive index of H_2_O in **Figure** **5**a implies a fingerprint peak feature at the wavelength of ≈6.06 µm.^[^ ^49^ ^]^ According to this feature, we apply the Metaformer on the smart design. Here, we adopt the user‐defined multiple dots to generate a target spectrum with a peak around the wavelength of 6.06 µm and input it to the Metaformer to achieve a real‐time design of metasensor structure parameters listed in Figure 5a.^[^ ^32^, ^39^ ^]^ It is worth mentioning that the metastructure parameters should consider the slight spectral shift due to the introduction of trace analyte coating during the design. By the flexible design tool, the metasensor resonance of Q‐BIC 1 mode can match the fingerprint feature of H_2_O very well, and the maximum enhancement of fingerprint absorbance can be optimized, as illuminated in Figure 5a. In fact, there is no obvious peak on the absorbance spectrum for the trace H_2_O detection on an unpatterned substrate of SiO_2_, which could be easily interfered by signal noise in practice. The fingerprint peak intensity for metasensing is 55 times of that for unpatterned detection. To analyze the enhancement mechanism, we calculate the distribution of absorbed power P_abs_ = 1/2ωε″|*E*|^2^, where ω is the angular frequency of incident light, ε″ is the imaginary part of the permittivity, and |*E*| denotes the localized electric field intensity.^[^ ^61^ ^]^ It is observed that most of the absorbed power is concentrated along the edges of four‐square patches in the meta‐atom, particularly along the patch edges along the *y*‐axis. Such an absorbed power distribution denotes the strong interaction between the IR light and trace H_2_O layer, which is induced by Q‐BIC 1 and leads to significant enhancement on trace fingerprint signal.

Furthermore, our tool can also design a fingerprint metasensor and utilize both Q‐BIC 1 and Q‐BIC 2 for the detection of trace analyte with two IR featured peaks, such as goat anti‐mouse immunoglobulin G (IgG),^[^ ^62^ ^]^ which has two distinct fingerprint features at the wavelengths of 6.02 and 6.55 µm denoting amide I and amide II of this protein, respectively, as shown in Figure 5b. For the detection of IgG with a thickness of 30 nm, the designed metasensor demonstrates two absorbance peaks ≈6.03 and 6.56 µm, respectively, which have a good match with the dual fingerprint features. Compared with the unpatterned detection, the enhancement factors of two fingeprint absorbance peaks are up to 51 and 95, respectively. They correspond to the localized enhancement of absorbance power on the metasensors and are attributed to the near‐field effects of Q‐BIC 1 and Q‐BIC 2, respectively, as observed in Figure 5b. The above results reveal the powerful function of our Metaformer on fingerprint metasensor design. In particular, one can introduce more design degrees of freedom, such as more asymmetric factors of Q‐BIC, in order to meet the metasensing demand for multiple fingerprint peak features. On this condition, one can also extend the metasensing design applications to various trace molecules.

### 2.4 Metasensor Design for RI Sensing

**Figure 6:**
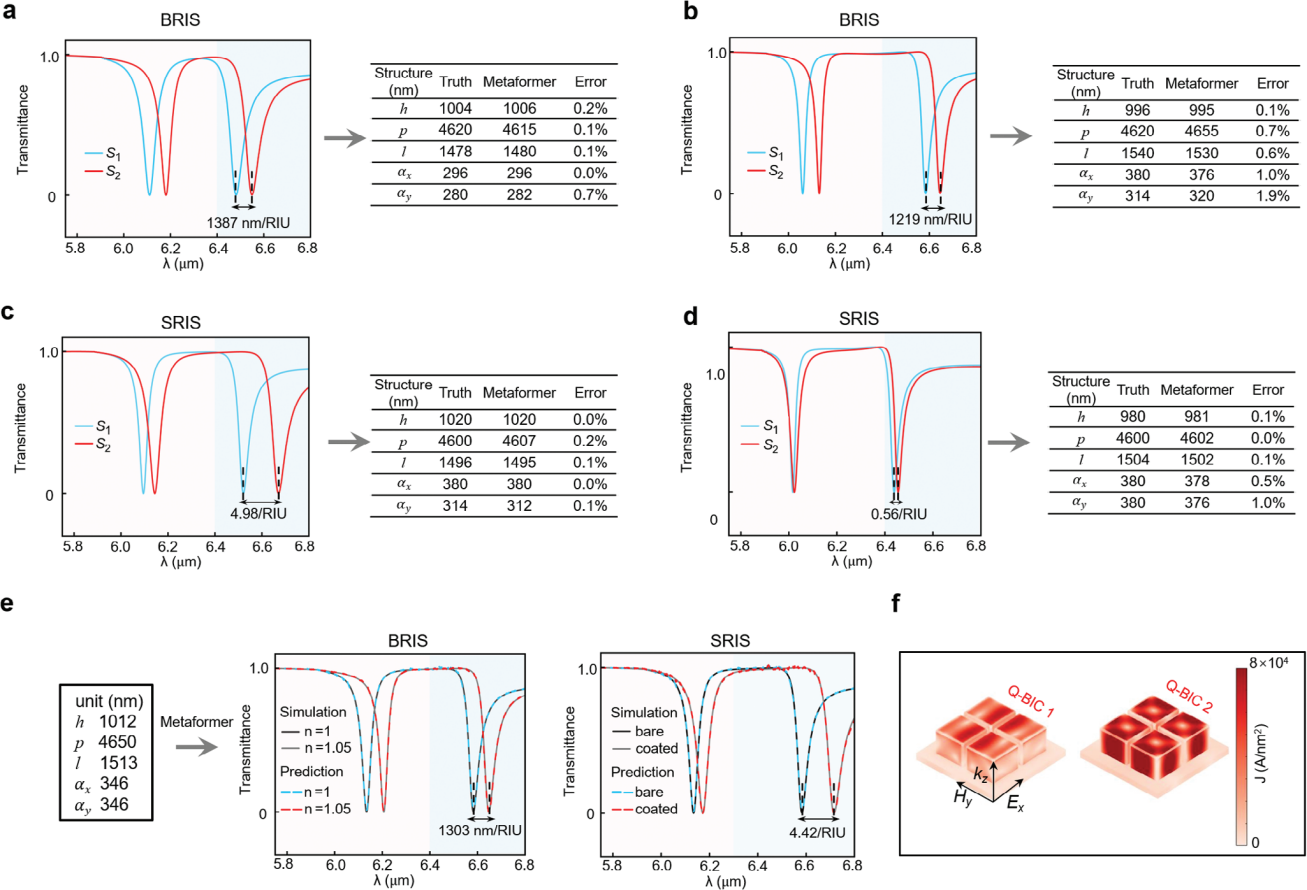
(a) and (b) are the inverse design for two given BRIS values. (c) and (d) are the inverse design for two given SRIS values. (e) is the forward design of BRIS and SRIS for a given set of metastructure parameters. f) The 3D displacement current density distributions of meta‐atom for the wavelengths of Q‐BIC 1 and Q‐BIC 2, respectively.

The Metaformer is also used for the inverse and forward design of metasensor with RI sensitivity, which is an important indicator of the detection performance. In the inverse process, we design the metastructure parameters according to the user‐defined optical sensitivity. In **Figure** **6**a,b, we pick up two testing instances with the BRIS values of 1387 and 1219 nm/RIU, respectively. The blue and red spectra correspond to the transmittance under the environmental refractive index values of *n* = 1 and 1.05, respectively. At the beginning, we adopt the user‐defined multiple dots to generate a spectrum under the air background of *n* = 1. According to the given BRIS value, the shifted spectrum due to environmental change of *n* = 1.05 can be obtained by appropriately moving the former spectrum toward longer wavelengths. After that, the two spectra are used as the input of the Metaformer to generate a predicted metastructure, which exhibits the largest relative error no more than 1.9% of the ground truth for its all five geometry parameters. In Figure 6c,d, we provide the demand of two testing samples for the trace metasensing of 20 nm poly(allylamine hydrochloride) (PAH)/poly(styrene sulfonate)(PSS) whose SRIS values are 4.98 and 0.56/RIU, respectively. Although the two SRIS values have a relatively big numerical difference, the Metaformer model can accurately predict their corresponding metastructure parameters with the largest relative error no more than 1.0% of the ground truth for all the five geometry sizes. These results indicate that one can freely customize a metasensor structure by a given target sensitivity, which could meet a variety of sensing criteria in many application strategies.^[^ ^62^, ^63^ ^]^ Besides, the forward design is quite essential to assess the BRIS and SRIS performance, once the geometry of a metastructure is provided. In Figure 6e, we show a set of randomly given structure parameters. When it is input into the Metaformer, we can obtain the corresponding spectra in different environments along with both the BRIS and SRIS in real time (see more details in Figure S7, Supporting Information). These results demonstrate that the Metaformer model can accurately predict the BRIS and SRIS for a given metasensor structure. Particularly, among the testing samples, we notice that the BRIS values of Q‐BIC 1 and Q‐BIC 2 for a given metasensor are very close, while their SRIS values have a relatively significant difference. In order to explain this, we plot the 3D surface profiles of displacement current *J* in Figure 6f.^[^ ^64^ ^]^ It can be observed that the meta‐atom surface current density of Q‐BIC 2 mode is markedly higher than that of Q‐BIC 1 mode, which leads to the larger SRIS and better surface sensing performance (see more details in Figure S8, Supporting Information). These results in Figure 6 exhibit the powerful capability of Metaformer in the smart inverse and forward design of RI metasensors, which could be well utilized for fast customized sensing development and smart sensitivity analysis of given sensors. Based on the above study, we further develop a flexible and universal tool that allows for quickly designing metasensors with different requirements (see more details in Figure S9, Supporting Information and the attached.gif movie files).

### 2.5 Metasensor Experiments Guided by the Design of Metaformer

**Figure 7:**
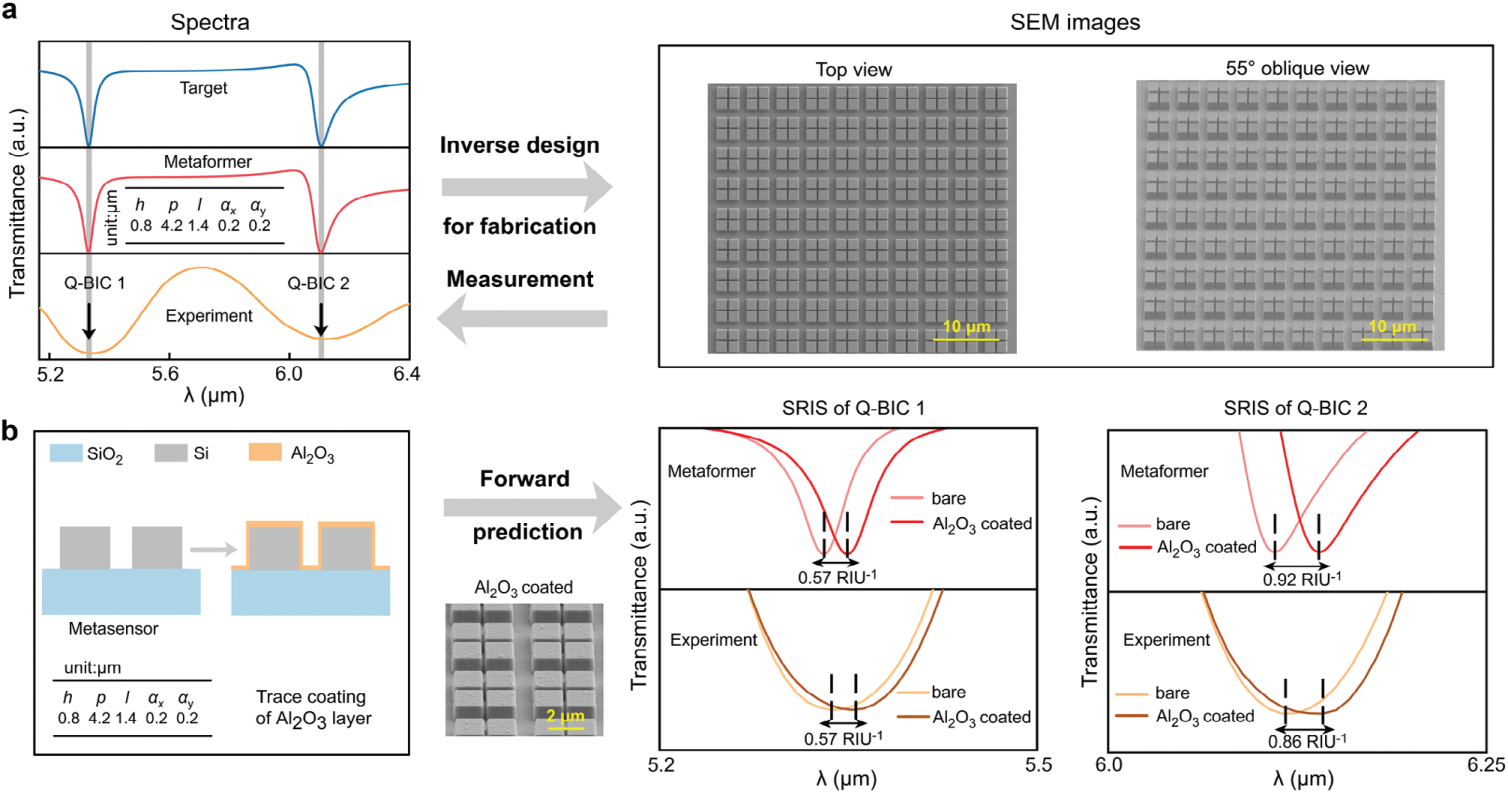
a) Inverse design for the fabrication of a metasurface for a given target spectrum with two featured valleys at the wavelengths of 5.3 and 6.1 µm, respectively, where the microstructure SEM images and measured spectrum are provided. b) Forward design result for SRIS prediction of a given metasensor structure compared to the experimental results, where the inset is the SEM images of Metasensor with Al_2_O_3_ by an oblique view.

Empowered by the Metaformer, we design and fabricate the metasurface for a given target spectrum with two featured valleys, followed by the microstructure characterization and optical measurement, as illustrated in **Figure** **7**a. The SEM images demonstrate that the fabricated metasurface has the uniform periodic arrays of meta‐atom with four square patches. In fact, the fabrication tolerance, variation of material optical parameters, and measurement of Fourier transform infrared (FTIR) could influence the experimental spectrum result, leading to smaller Q factors of the two measured Q‐BIC resonances than those designed by the Metaformer.^[^ ^65^ ^]^ Despite this, the measured spectrum demonstrates good consistency to that from the Metaformer, especially for the resonance feature locations of Q‐BIC 1 and Q‐BIC 2, which implies our design tool should have a good potential to guide the fabrication of fingerprint metasensor. Furthermore, we use the Metaformer to predict the SRIS values of the two Q‐BIC resonances for a given set of metasensor structure parameters and validate it with the experimental result in Figure 7b. The measured SRIS values along with the corresponding spectra before and after the coating of a trace Aluminium oxide (Al_2_O_3_) layer indicate good agreement with those from the design result of Metaformer. This result suggests that one can assess the sensitivity performance of a given metasensor in real time, avoid redundant experiments and speed the device development efficiency. In general, the experimental work aided by the Metaformer elucidates that the learning‐explainable transformer architecture is highly efficient for intelligent design of Q‐BIC‐based metasensors.

## 3 Conclusion

In summary, we establish a transformer‐based deep learning model for the quick smart design of Q‐BIC metasensors, which is promising for optical fingerprint and RI detection. The model can effectively capture the high‐Q spectral features, enabling higher prediction precision on sensing performance by decreasing the training parameters of 99% than conventional approaches in the inverse design. We study a series of critical component stages within the network and explain the powerful learning performance of the model. On this basis, we develop a flexible tool allowing for fast design of both fingerprint metasensors and RI metasensors. Empowered by the learning model, we further fabricate and measure the Q‐BIC metasensor as a proof‐of‐concept example. Our scheme gives a powerful guideline for intelligent design of metasensors and will also facilitate more applications in the field of metasurfaces and metadevices.

## 4 Experimental Section

### Sample Preparation

The workflow of metasurface fabrication is shown in Figure S10 (Supporting Information). Initially, a silicon layer of 1000 nm thick is deposited on the 1 × 1 cm quartz substrate by magnetron sputtering with the rate of 2.8 Å s^−1^ (JC‐500‐3/D, Chengdu Vacuum Machinery Factory, China). The power and Ar flow rate are 200 W and 100 sccm, respectively. Next, a polymethylmethacrylate (PMMA) resist film of 150 nm thick (950k, Allresist, Germany) is spin‐coated on the substrate and exposed for the meta‐atom pattern by electron beam lithography (Sigma 300 and ELPHY Quantum, Carl Zeiss, Germany). After the development of resist, a 20 nm chromium film is evaporated on the patterned PMMA at the rate of 0.1 nm s^−1^ (DE400, DE Technology Inc., Germany), which is followed by the lift‐off process of PMMA and keeps the chromium hard mask of meta‐atom pattern. The sample is next placed in an inductively coupled plasma system to etch silicon for 5 min, where the reactive gases are O_2_, SF_6_, and Ar. Finally, the chromium hard mask is removed by its etchant, by which the ultimate metasurface is achieved. In order to evaluate the surface refractive index sensitivity of the metasurface, a dense thin film of 20 nm Al_2_O_3_ is conformally coated on its surface by atomic layer deposition at the rate of 0.1 nm s^−1^ (TFS‐200‐PEALD, Beneq, Finland; see more details in Figure S11, Supporting Information).

### Characterizations and Measurements

The sample morphology is characterized by the dual‐beam scanning electron microscope (Solaris, Tescan, Czech Republic). The top view and tilted view characterizations of the sample is achieved by setting the pitch angles of translation stage to 0° and 55°. In the optical measurement, the microscopic spectra are obtained by using the Fourier transform infrared spectrometer (LUMOS II, Bruker, Germany). For each metasensor sample, 20 microscopic spectra are recorded, averaged, and normalized.

### Modeling and Simulations

The optical simulation of metasurfaces is performed by electromagnetic full‐wave software Ansys Lumerical, which is based on the Finite‐Difference Time‐Domain (FDTD) algorithm. The optical waves are incident vertically on the metasensors with the electric polarization along the *x* direction. The periodic boundary conditions along *x*‐axis and *y*‐axis, as well as the perfectly matched layers along *z*‐axis are applied on the unit cell of meta‐atom to simulate the optical responses of metasurfaces. The mesh is configured based on a self‐adapted scheme to ensure the accuracy of numerical calculation. During the simulation, the values of five parameters, including *p*, *l*, *h*, *α*_x_, and *α*_y_, are randomly changed within the defined ranges listed in Table S3 (Supporting Information). Based on the simulation, three groups of data, which corresponds to three different dielectric environments of the metasurfaces, were collected. The three groups denote the background refractive index *n* = 1, the background refractive index *n* = 1.05, and the surface layer coating of 20 nm alternating polyelectrolytes, corresponding to the investigation of bare metasensors, BRIS and SRIS, respectively.

### Data Collection and Training Process

Twenty nine thousand four hundred samples for each group are randomly collected by the software Lumerical FDTD solutions. The simulated data are shuffled, and 80% are blindly selected as the training samples, the remaining 20% are used for validation and testing samples. The structure parameters dimensions are 1 × 5 and the spectrum dimensions are 1 × 1000. The head of transformer is 10 and the layer of transformer is 10 for inverse design. The head of transformer is 4 and the layer of transformer is 3 for forward design. d*_k_* is 64 and the learning rate is 0.001. The Metaformer model are trained using Python version 3.8 and the open‐source deep learning framework of PyTorch on a local computer (Windows10 operation system, GeForce GTX 3080Ti GPU, Intel(R) Core (TM) i7‐10700K CPU @ 3.80 GHz 3.79 GHz, and 16GB of RAM.). It takes nearly 5 h for each network to converge after running 1000 epochs using a batch size of 256.

## Conflict of Interest

The authors declare no conflict of interest.

## Supporting information



Supporting Information

Supporting Information

Supporting Information

Supporting Information

## Data Availability

The data that support the findings of this study are available from the corresponding author upon reasonable request.

## References

[1] Y. Chen , H. Deng , X. Sha , W. Chen , R. Wang , Y. H. Chen , D. Wu , J. Chu , Y. S. Kivshar , S. Xiao , C. W. Qiu , Nature 2023, 613, 474. 36653568 10.1038/s41586-022-05467-6

[2] X. Zhang , Y. Liu , J. Han , Y. Kivshar , Q. Song , Science 2022, 377, 1215. 36074858 10.1126/science.abq7870

[3] B. Xiong , Y. Liu , Y. Xu , L. Deng , C.‐W. Chen , J.‐N. Wang , R. Peng , Y. Lai , Y. Liu , M. Wang , Science 2023, 379, 294. 36656947 10.1126/science.ade5140

[4] F. Li , J. Shen , C. Guan , Y. Xie , Z. Wang , S. Lin , J. Chen , J. Zhu , Biosens. Bioelectron. 2022, 203, 114038. 35121450 10.1016/j.bios.2022.114038

[5] A. Tittl , A. Leitis , M. Liu , F. Yesilkoy , D.‐Y. Choi , D. N. Neshev , Y. S. Kivshar , H. Altug , Science 2018, 360, 1105. 29880685 10.1126/science.aas9768

[6] Y. Chen , W. Chen , X. Kong , D. Wu , J. Chu , C. W. Qiu , Phys. Rev. Lett. 2022, 128, 146102. 35476494 10.1103/PhysRevLett.128.146102

[7] D. Kavungal , P. Magalhães , S. T. Kumar , R. Kolla , H. A. Lashuel , H. Altug , Sci. Adv. 2023, 9, eadg9644. 37436975 10.1126/sciadv.adg9644 PMC10337894

[8] Z. Dong , L. Jin , S. D. Rezaei , H. Wang , Y. Chen , F. Tjiptoharsono , J. Ho , S. Gorelik , R. J. H. Ng , Q. Ruan , C.‐W. Qiu , J. K. W. Yang , Sci. Adv. 2022, 8, eabm4512. 35196088 10.1126/sciadv.abm4512 PMC8865777

[9] Z. Dong , Z. Mahfoud , R. Paniagua‐Domínguez , H. Wang , A. I. Fernández‐Domínguez , S. Gorelik , S. T. Ha , F. Tjiptoharsono , A. I. Kuznetsov , M. Bosman , J. K. W. Yang , Light Sci. Appl. 2022, 11, 20. 35058424 10.1038/s41377-021-00707-2 PMC8776833

[10] A. Leitis , M. L. Tseng , A. John‐Herpin , Y. S. Kivshar , H. Altug , Adv. Mater. 2021, 33, 2102232. 34494318 10.1002/adma.202102232 PMC11468586

[11] J. Zhu , Z. Wang , S. Lin , S. Jiang , X. Liu , S. Guo , Biosens. Bioelectron. 2020, 150, 111905. 31791874 10.1016/j.bios.2019.111905

[12] D. Rodrigo , A. Tittl , N. Ait‐Bouziad , A. John‐Herpin , O. Limaj , C. Kelly , D. Yoo , N. J. Wittenberg , S. H. Oh , H. A. Lashuel , H. Altug , Nat. Commun. 2018, 9, 2160. 29867181 10.1038/s41467-018-04594-x PMC5986821

[13] F. Jiao , F. Li , J. Shen , C. Guan , S. A. Khan , J. Wang , Z. Yang , J. Zhu , Sens. Actuators B: Chem. 2021, 344, 130170.

[14] A. Belushkin , F. Yesilkoy , H. Altug , ACS Nano 2018, 12, 4453. 29715005 10.1021/acsnano.8b00519

[15] S. H. Oh , H. Altug , Nat. Commun. 2018, 9, 5263. 30531967 10.1038/s41467-018-06419-3 PMC6288137

[16] D. Hasan , C. Lee , Adv. Sci. 2018, 5, 1700581. 10.1002/advs.201700581 PMC5978960 29876204

[17] K. V. Sreekanth , S. Sreejith , S. Han , A. Mishra , X. Chen , H. Sun , C. T. Lim , R. Singh , Nat. Commun. 2018, 9, 369. 29371614 10.1038/s41467-018-02860-6 PMC5785542

[18] I. Malkiel , M. Mrejen , A. Nagler , U. Arieli , L. Wolf , H. Suchowski , Light Sci. Appl. 2018, 7, 60. 30863544 10.1038/s41377-018-0060-7 PMC6123479

[19] Y. LeCun , Y. Bengio , G. Hinton , Nature 2015, 521, 436. 26017442 10.1038/nature14539

[20] J. Jumper , R. Evans , A. Pritzel , T. Green , M. Figurnov , O. Ronneberger , K. Tunyasuvunakool , R. Bates , A. Zidek , A. Potapenko , A. Bridgland , C. Meyer , S. A. A. Kohl , A. J. Ballard , A. Cowie , B. Romera‐Paredes , S. Nikolov , R. Jain , J. Adler , T. Back , S. Petersen , D. Reiman , E. Clancy , M. Zielinski , M. Steinegger , M. Pacholska , T. Berghammer , S. Bodenstein , D. Silver , O. Vinyals , et al., Nature 2021, 596, 583. 34265844 10.1038/s41586-021-03819-2 PMC8371605

[21] S. Noy , W. Zhang , Science 2023, 381, 187. 37440646 10.1126/science.adh2586

[22] W. Ma , Z. Liu , Z. A. Kudyshev , A. Boltasseva , W. Cai , Y. Liu , Nat. Photonics 2020, 15, 77.

[23] S. Krasikov , A. Tranter , A. Bogdanov , Y. Kivshar , Opto‐Electron. Adv. 2022, 5, 210147.

[24] C. Liu , Q. Ma , Z. J. Luo , Q. R. Hong , Q. Xiao , H. C. Zhang , L. Miao , W. M. Yu , Q. Cheng , L. Li , T. J. Cui , Nat. Electron 2022, 5, 113.

[25] W. Chen , J. Shen , Y. Ke , Y. Hu , L. Wu , J. Zhu , Z. Domg , World Sci. Ann. Rev. Funct. Mater. 2024, 0, 2430002.

[26] Y. Chen , J. Zhu , Y. Xie , N. Feng , Q. H. Liu , Nanoscale 2019, 11, 9749. 31066432 10.1039/c9nr01315f

[27] X. Han , Z. Fan , Z. Liu , C. Li , L. J. Guo , InfoMat 2020, 3, 432.

[28] J. Jiang , J. A. Fan , Nano Lett. 2019, 19, 5366. 31294997 10.1021/acs.nanolett.9b01857

[29] E. Khoram , Z. Wu , Y. Qu , M. Zhou , Z. Yu , ACS Photonics 2022, 10, 892.

[30] W. Ma , F. Cheng , Y. Xu , Q. Wen , Y. Liu , Adv. Mater. 2019, 31, 1901111. 10.1002/adma.201901111 31259443

[31] C. C. Nadell , B. Huang , J. M. Malof , W. J. Padilla , Opt. Express 2019, 27, 27523. 31684518 10.1364/OE.27.027523

[32] J. Xiong , J. Shen , Y. Gao , Y. Chen , J. Y. Ou , Q. H. Liu , J. Zhu , Laser Photon. Rev. 2022, 17, 2100738.

[33] T. Li , A. Chen , L. Fan , M. Zheng , J. Wang , G. Lu , M. Zhao , X. Cheng , W. Li , X. Liu , H. Yin , L. Shi , J. Zi , Light Sci. Appl. 2021, 10, 154. 34315850 10.1038/s41377-021-00600-y PMC8316458

[34] J. Y. Jia , C. Qian , Z. Fan , T. Cai , E. P. Li , H. Chen , Light Sci. Appl. 2023, 12, 82. 36997520 10.1038/s41377-023-01131-4 PMC10060944

[35] W. Chen , Y. Li , Y. Liu , Y. Gao , Y. Yan , Z. Dong , J. Zhu , Adv. Opt. Mater 2023, 12, 2301697.

[36] R. Zhu , T. Qiu , J. Wang , S. Sui , C. Hao , T. Liu , Y. Li , M. Feng , A. Zhang , C. W. Qiu , S. Qu , Nat. Commun. 2021, 12, 2974. 34016963 10.1038/s41467-021-23087-y PMC8137937

[37] W. Ma , W. Chen , D. Li , Y. Liu , J. Yin , C. Tu , Y. Xia , G. Shen , P. Zhou , L. Deng , L. Zhang , Nanophotonics 2023, 12, 3589. 10.1515/nanoph-2023-0291 PMC11502052 39635349

[38] D. Piccinotti , K. F. MacDonald , A. G. S. , I. Youngs , N. I. Zheludev , Rep. Prog. Phys. 2021, 84, 012401. 33355315 10.1088/1361-6633/abb4c7

[39] W. Chen , Y. Gao , Y. Li , Y. Yan , J. Y. Ou , W. Ma , J. Zhu , Adv. Sci. 2023, 10, 2206718. 10.1002/advs.202206718 PMC10161039 36852630

[40] Y. Li , Z. Zhou , C. Sun , X. Chen , R. Yan , IEEE Transact. Neural Networks Learning Syst. 2024, 35, 6180. 10.1109/TNNLS.2022.3202234 36094988

[41] M. Castangia , L. M. M. Grajales , A. Aliberti , C. Rossi , A. Macii , E. Macii , E. Patti , Environ. Modelling Software 2023, 160, 105581.

[42] H. Liang , X. Wang , F. Li , Y. Xie , J. Shen , X. Wang , Y. Huang , S. Lin , J. Chen , L. Zhang , B. Jiang , J. Xing , J. Zhu , Biosens. Bioelectron. 2023, 235, 115380. 37207584 10.1016/j.bios.2023.115380

[43] F. Li , J. Hong , C. Guan , K. Chen , Y. Xie , Q. Wu , J. Chen , B. Deng , J. Shen , X. Liu , R. Hu , Y. Zhang , Y. Chen , J. Zhu , ACS Nano 2023, 17, 3383. 36630157 10.1021/acsnano.2c08153

[44] K. He , X. Zhang , S. Ren , J. Sun , in IEEE Conference on Computer Vision and Pattern Recognition (CVPR), Las Vegas, NV, USA 2016, 770.

[45] A. Vaswani , N. Shazeer , N. Parmar , J. Uszkoreit , L. Jones , N. Gomez , L. Kaiser , in Proceedings of the 31st International Conference on Neural Information Processing Systems, CA, USA 2017, 6000.

[46] J. Jin , X. Yin , L. Ni , M. Soljačić , B. Zhen , C. Peng , Nature 2019, 574, 501. 31645728 10.1038/s41586-019-1664-7

[47] V. Ardizzone , F. Riminucci , S. Zanotti , A. Gianfrate , M. Efthymiou‐Tsironi , D. G. Suàrez‐Forero , F. Todisco , M. De Giorgi , D. Trypogeorgos , G. Gigli , K. Baldwin , L. Pfeiffer , D. Ballarini , H. S. Nguyen , D. Gerace , D. Sanvitto , Nature 2022, 605, 447. 35585343 10.1038/s41586-022-04583-7

[48] X. Liu , W. Chen , Y. Ma , Y. Xie , J. Zhou , L. Zhu , Y. Xu , J. Zhu , Photon. Res. 2022, 10, 2836.

[49] W. M. Haynes , D. R. Lide , in CRC Handbook of Chemistry and Physics: A Ready‐Reference Book of Chemical and Physical Data, CRC Press, Boca Raton 2010.

[50] K. Koshelev , S. Lepeshov , M. Liu , A. Bogdanov , Y. Kivshar , Phys. Rev. Lett. 2018, 121, 193903. 30468599 10.1103/PhysRevLett.121.193903

[51] M. F. Limonov , M. V. Rybin , A. N. Poddubny , Y. S. Kivshar , Nat. Photonics 2017, 11, 543.

[52] J. Wang , J. Kühne , T. Karamanos , C. Rockstuhl , S. A. Maier , A. Tittl , Adv. Funct. Mater. 2021, 31, 2104652.

[53] A. A. Bogdanov , A. Fratalocchi , Y. Kivshar , Nanophotonics 2021, 10, 4171.

[54] I. Beltagy , M. E. Peters , A. Cohan , arXiv preprint arXiv:2004.05150 , 2020.

[55] N. Kitaev , Ł. Kaiser , A. Levskaya , in International Conference on Learning Representations (ICLR), 2020.

[56] A. Dosovitskiy , L. Beyer , A. Kolesnikov , D. Weissenborn , X. Zhai , T. Unterthiner , M. Dehghani , M. Minderer , G. Heigold , S. Gelly , J. Uszkoreit , N. Houlsby , in International Conference on Learning Representations (ICLR), 2021.

[57] J. Peurifoy , Y. Shen , L. Jing , Y. Yang , F. Cano‐Renteria , B. G. DeLacy , J. D. Joannopoulos , M. Tegmark , M. Soljačić , Sci. Adv. 2018, 4, eaar4206. 29868640 10.1126/sciadv.aar4206 PMC5983917

[58] J. Chen , C. Qian , J. Zhang , Y. Jia , H. Chen , Nat. Commun. 2023, 14, 4872. 37573442 10.1038/s41467-023-40619-w PMC10423275

[59] D. Liu , Y. Tan , E. Khoram , Z. Yu , ACS Photonics 2018, 5, 1365.

[60] W. Ma , F. Cheng , Y. Liu , ACS Nano 2018, 12, 6326. 29856595 10.1021/acsnano.8b03569

[61] J. Liu , W.‐Z. Ma , W. Chen , Y.‐S. Chen , X.‐C. Deng , Y. Gu , IEEE Access 2021, 9, 92941.

[62] M. Ye , K. B. Crozier , Opt. Express 2020, 28, 18479. 32680046 10.1364/OE.394564

[63] A. Lochbaum , A. Dorodnyy , U. Koch , S. M. Koepfli , S. Volk , Y. Fedoryshyn , V. Wood , J. Leuthold , Nano Lett. 2020, 20, 4169. 32343585 10.1021/acs.nanolett.0c00483

[64] Y. Wang , Z. Han , Y. Du , J. Qin , Nanophotonics 2021, 10, 1295.

[65] S. Yang , M. He , C. Hong , J. D. Caldwell , J. C. Ndukaife , Nano Lett. 2022, 22, 8060. 36214538 10.1021/acs.nanolett.2c01919

