# Supplementary material for: Meta‐Attention Deep Learning for Smart Development of Metasurface Sensors
Source: Adv Sci (Weinh). 2024 Sep 9;11(42):2405750. doi: 10.1002/advs.202405750 (PMC11558086; doi:10.1002/advs.202405750)
Supplement: Supplementary file 1 — Supporting Information [file ADVS-11-2405750-s002.pdf]

ADVANCED SCIENCE
Open Access

Supporting Information

for *Adv. Sci.*, DOI 10.1002/advs.202405750

Meta-Attention Deep Learning for Smart Development of Metasurface Sensors

*Yuan Gao, Wei Chen, Fajun Li, Mingyong Zhuang, Yiming Yan, Jun Wang, Xiang Wang, Zhaogang Dong, Wei Ma and Jinfeng Zhu**

Supporting Information

Meta-attention deep learning for smart development of metasurface sensors

Yuan Gao[1, #], Wei Chen[1, #], Fajun Li[1, #], Mingyong Zhuang[1], Yiming Yan[1], Jun Wang[2], Xiang Wang[2], Zhaogang Dong[3,4], Wei Ma[5], Jinfeng Zhu[1, *]

[1] Institute of Electromagnetics and Acoustics and Key Laboratory of Electromagnetic Wave Science and Detection Technology, Xiamen University, Xiamen, Fujian 361005, China

[2] State Key Laboratory of Physical Chemistry of Solid Surfaces, Department of Chemistry, College of Chemistry and Chemical Engineering, Xiamen University, Xiamen 361005, China

[3] Institute of Materials Research and Engineering (IMRE), Agency for Science, Technology and Research (A*STAR), 2 Fusionopolis Way, Innovis # 08-03, Singapore 138634, Republic of Singapore

[4] Department of Materials Science and Engineering, National University of Singapore, 9 Engineering Drive 1, 117575, Singapore

[5] College of Information Science and Electronic Engineering, Zhejiang University, Hangzhou 310027, China

# These authors contributed equally

*E-mail: jfzhu@xmu.edu.cn

Fano Formula Fitting

The Q-BIC spectral profile shows an asymmetric feature, which can be fitted by Fano resonance theory as below [1]:

$$R(\lambda) = 1 - T_0 - A_0 \frac{[q+2(\lambda-\lambda_0)/\tau]^2}{1+[2(\lambda-\lambda_0)/\tau]^2} \quad (1)$$

where T_0 is the transmission offset, A_0 is the continuum -discrete coupling constant, q is Breit-Wigner-Fano parameters and τ is the resonant linewidth, λ_0 is the resonance wavelength. We fit the simulated resonance curves of Q-BIC 1 and Q-BIC 2 in Figure 2c by using the above formula. T_0, A_0, q, λ_0, and τ are all unknowns, which need to be performed multiple iterative fittings to find the fitting coefficients that match the spectra.

Multipole Decomposition

To have a deep insight into the mechanism of two Q-BIC modes, we calculate the scattering cross sections of metasurface by the the multipole decomposition method. The normalized total scattering cross-section can be determined by the following equation [2],

$$C_{\mathrm{total}} = C_{\mathrm{ED}} + C_{\mathrm{MD}} + C_{\mathrm{EQ}} + C_{\mathrm{MQ}} \quad (2)$$

where the scattering cross-sections of different multipoles includes electric dipole (ED), magnetic dipole (MD), electric quadrupole (EQ) and magnetic quadrupole (MQ).

We compare the performance of our method with the MLP model, which is a kind of classic deep neural network. The layer sizes of inverse network are {1000, 1024, 1024, 1024, 1024, 1024, 1024, 1024,1024, 1024, 5}. The layer sizes of forward network (FNN) are {5, 2100, 2100, 2100, 2100, 2100, 2100, 2100, 2100, 2100, 1000}, as shown in Figure S1. We compare the forward and inverse network performance of the Metaformer model with the MLP model in **Table S1** and **Table S2**. Table S1 evaluates the accuracy for spectrum prediction based on the forward process of Metaformer and MLP. The Metaformer architecture indicates a prediction error reduction of 28% compared to the MLP model. Table S2 exhibits the prediction accuracy of metasensor structure parameters for the inverse process of Metaformer and MLP. Particularly, the result indicates an MSE error decrease of 44.8% by reducing 54.8% training parameters compared to the MLP model.

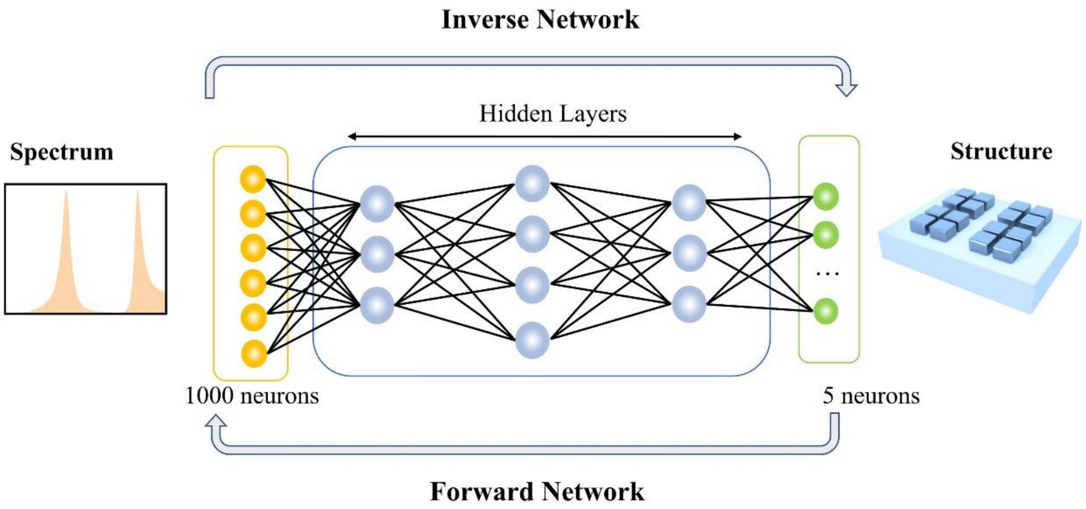

Figure S1 Schematic drawing of the MLP-enabled model for designing Metasensors.

Table S1 Forward design performance comparison between the MLP and Metaformer

Method	MSE	Training parameters
MLP	1.29×10^{-4}	37410400
Metaformer	9.3×10^{-5}	36319408
Critical reduction for improvement	28%	3%

Table S2 Inverse design performance comparison between the MLP and Metaformer

Method	MSE	Training parameters
MLP	2.9×10^{-6}	9426949
Metaformer	1.6×10^{-6}	4262173
Critical reduction for improvement	44.8%	54.8%

We can obtain the predicted structure parameters through the inverse network, and then input these predicted parameters into the forward network to get the predicted spectrum for verification. We randomly pick up six instances of inverse and forward design and provide a comparison with the ground truth and MLP prediction, as shown in **Figure S2**. For the inverse design, the improved prediction accuracy of Metaformer will result in smaller structure parameter errors compared to MLP model. The prediction error of structure parameters for MLP model will cause obvious resonance wavelength shift errors, as shown in Figure S2. The resonance wavelength is crucial for the metasensor design, whose shift errors will significantly impact the design performance prediction of optical sensing. Additionally, the significant structure parameters error of the MLP model can even cause spectrum distortions in Figure S2(d).

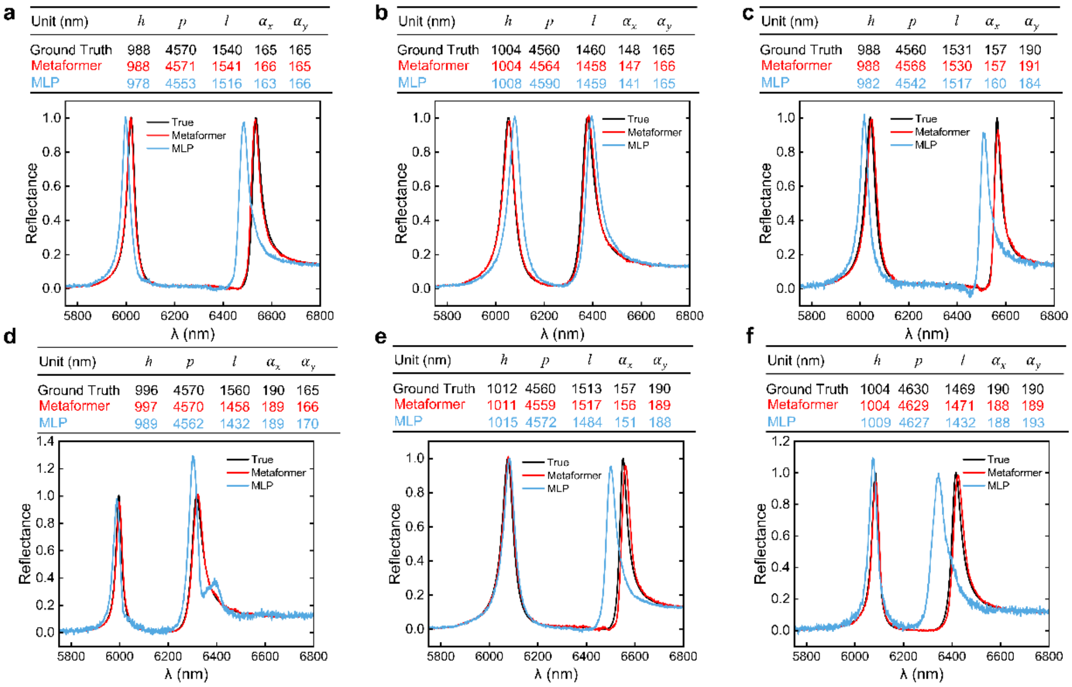

Figure S2 Six randomly selected instances of inverse and forward design from testing sample space.

We randomly pick two samples and count the percentage of different regions, respectively in Figure R3(a) and Figure R3(b), the percentage of sample points for two resonance regions is less than the other flat regions. DL is a method of data-driven artificial intelligence, so the sharp resonance with less sample points is harder to be learned than other flat parts. On the other hand, the strong variations around the resonance wavelengths also increase the prediction difficulty for DL model. It can be seen in Figure R3(c) that the MLP struggles to predict the true intensity

at the resonance positions. In Figure R3(d), there are also resonance positions shift errors. It is because that the resonance peaks occupy a smaller proportion of the sampling points than the other flat regions and have stronger variations around resonance wavelengths, which makes the prediction much more challenging.

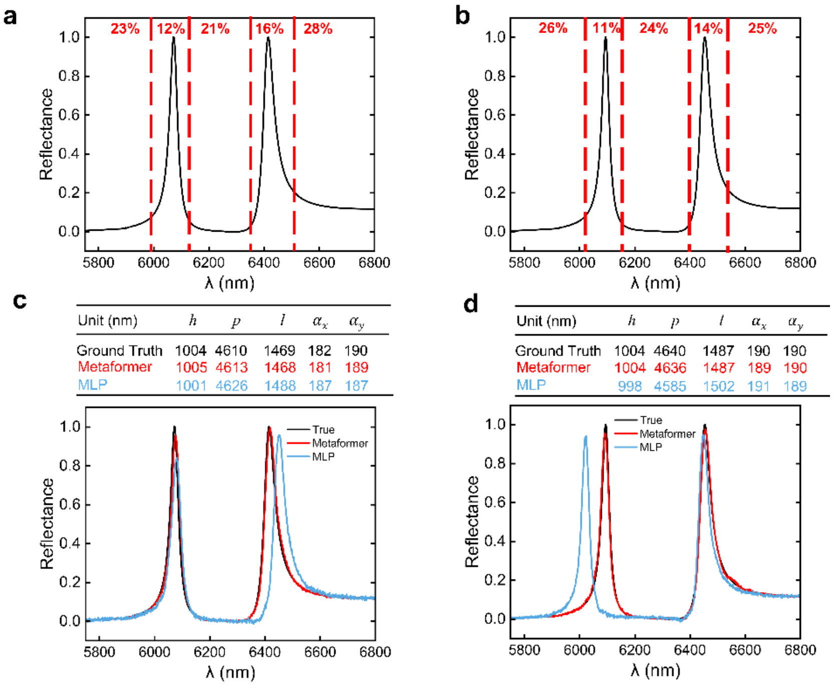

Figure S3 (a, b) Randomly spectral samples which exhibit percentage of sampling points occupied by the resonant and flat regions. (c, d) The inverse design and forward design for the samples in Figure R4 (a) and Figure R4 (b).

The entire Metaformer model adopts Adam as the gradient descent optimizer [3]. The ReLU function is used as an activation function [4]. We plot the learning accuracy as a function of M values in **Figure S4(a)**. To evaluate our method in detail, we randomly pick up a testing instance and analyze its prediction result in Figure S1b. The heatmap of position encoding matrix is shown in Figure S1(c).

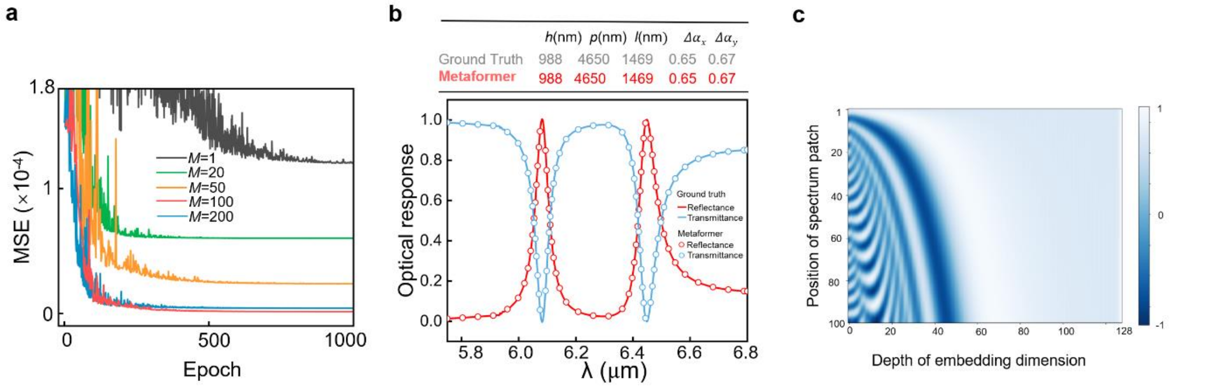

Figure S4 (a) Learning curves for different *patch* values in the inverse design. (b) Randomly selected testing instance of metasensor in the inverse design. (c) The heatmap of position encoding matrix.

DNNs are typically regarded as "black boxes", because it is difficult to interpret how a DNN makes a decision [5]. We plot the heatmaps of the weights associated with a neuron between adjacent layers in a NN as shown in **Figure S5**. Its results could not be obvious to exhibit the explainable phenomenon that can help us to understand the learning mechanism of DL model. With the increase of non-linear aggregation layers to achieve more powerful learning capability, the NN gets deeper meanwhile the contribution from input gets harder to trace, which leads to the loss of interpretability. However, the Transformer framework does not involve dimensionality reduction, thus keeping all attention layer traceable to the original input features, thus making the models interpretable [6].

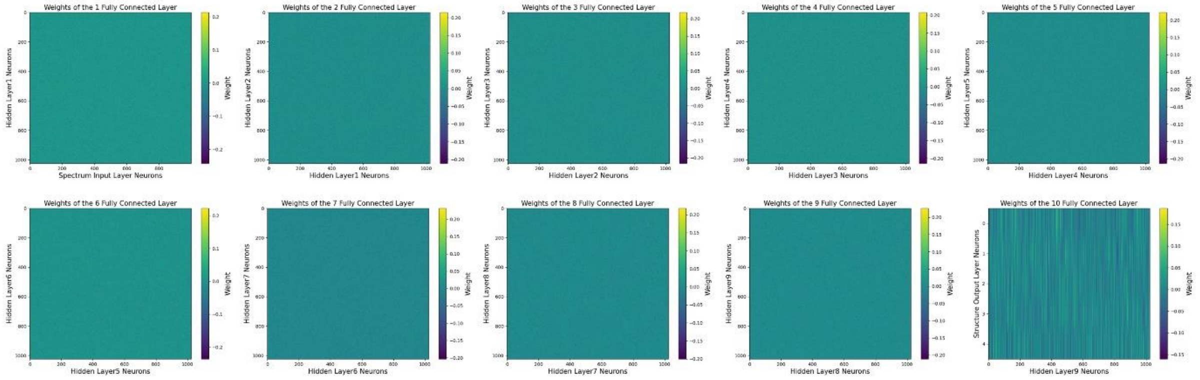

Figure S5 Heatmaps of the weights associated with a neuron between adjacent layers in a NN.

In order to have deeper understanding of the multi-head attention mechanism of Metaformer, we randomly select a sample from the testing space and plot the attention weight heatmaps for different layers and heads. **Figure S6** describes the attention weight heatmaps during inverse process. The attention weight visualization shows how the Metaformer learns different spectral feature details.

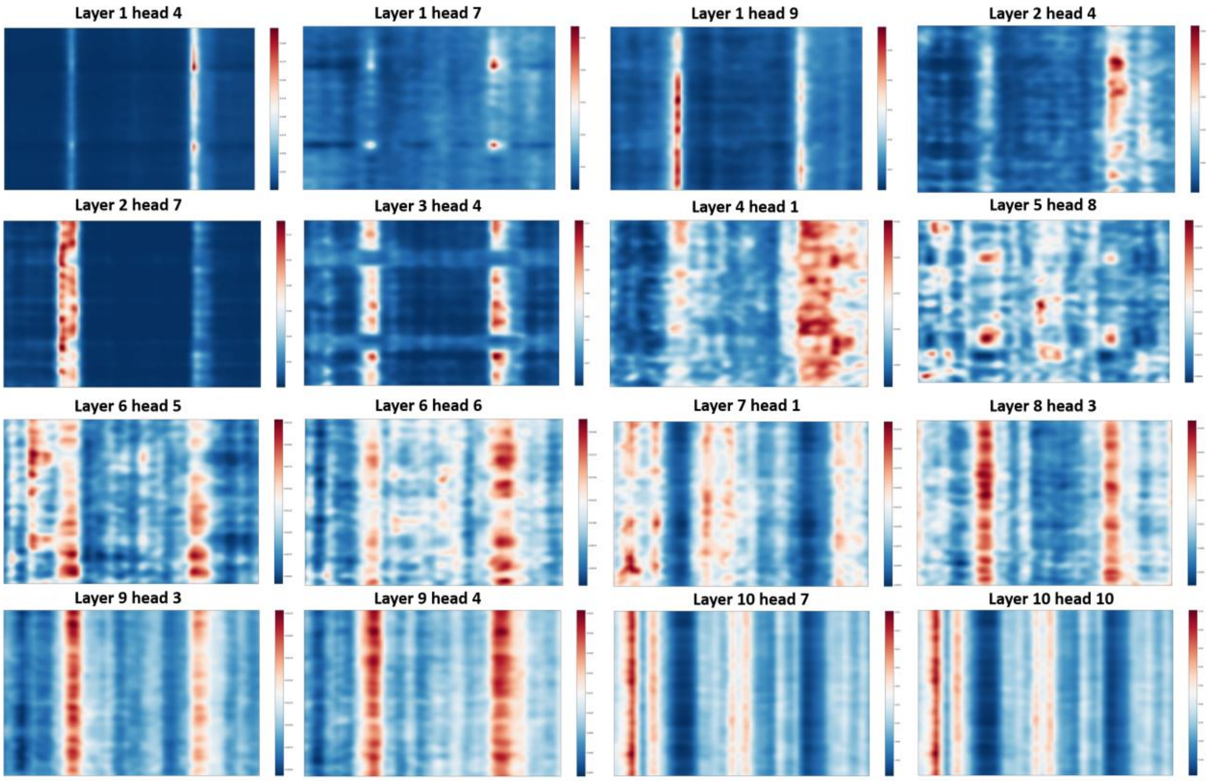

Figure S6 Heatmaps of attention weight for various network layers and multiple heads.

Based on the simulation, we collect three groups of data, which represent the conditions of the background refractive index *n*=1, the background refractive index *n*=1.05 and the surface layer coating of 20nm alternating polyelectrolytes. They correspond to the investigation of bare metasensor, BRIS and SRIS, respectively. We plot the learning curves of Metaformer for the three groups of data in **Figure S7(a)**. To evaluate the performance of BRIS and SRIS prediction, we randomly pick up two samples, as shown in Figure S7(b) and Figure S7(c).

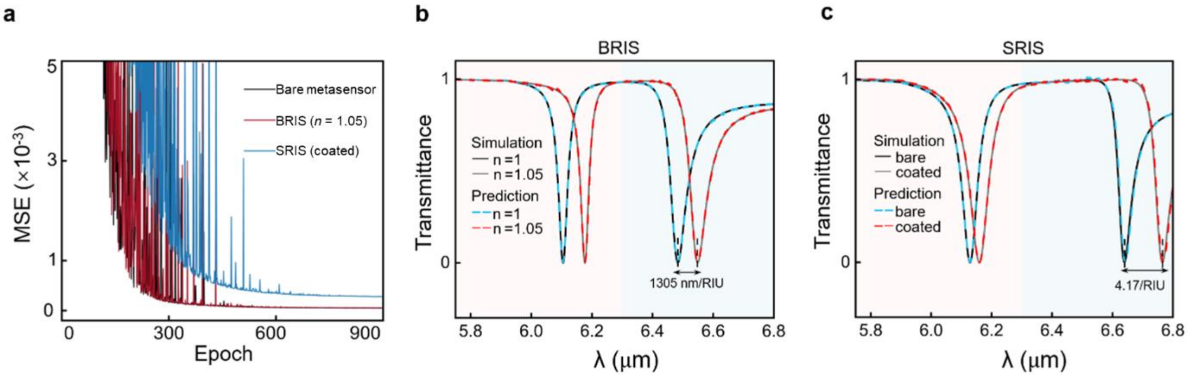

Figure S7 (a) Learning curves of Metaformer for three groups of data which corresponds to the investigation of bare metasensor, BRIS and SRIS in the forward design. (b,c) two Randomly-selected samples for sensitivity prediction by Metaformer from the testing samples.

To further explore the physical mechanisms between two Q-BIC modes, we calculate the current density J, and the electric field distributions of $|E/E_0|$ at resonance wavelengths, where E denotes localized electric field and E_0 is the amplitude of the incident field. **Figure S(a)** and Figure S8(b) show that two Q-BICs can be recognized as the collective response of four MDs due to the displacement currents' circular behavior surrounding each patch. The differences between the electric field at two resonances are highlighted from Figure S8(c) to Figure S8(f), demonstrating that one resonance is a combined result of the out-plane adjacent meta-atoms as well as in-plane four square patches, while another resonance is the result of in-plane coupling of four square patches.

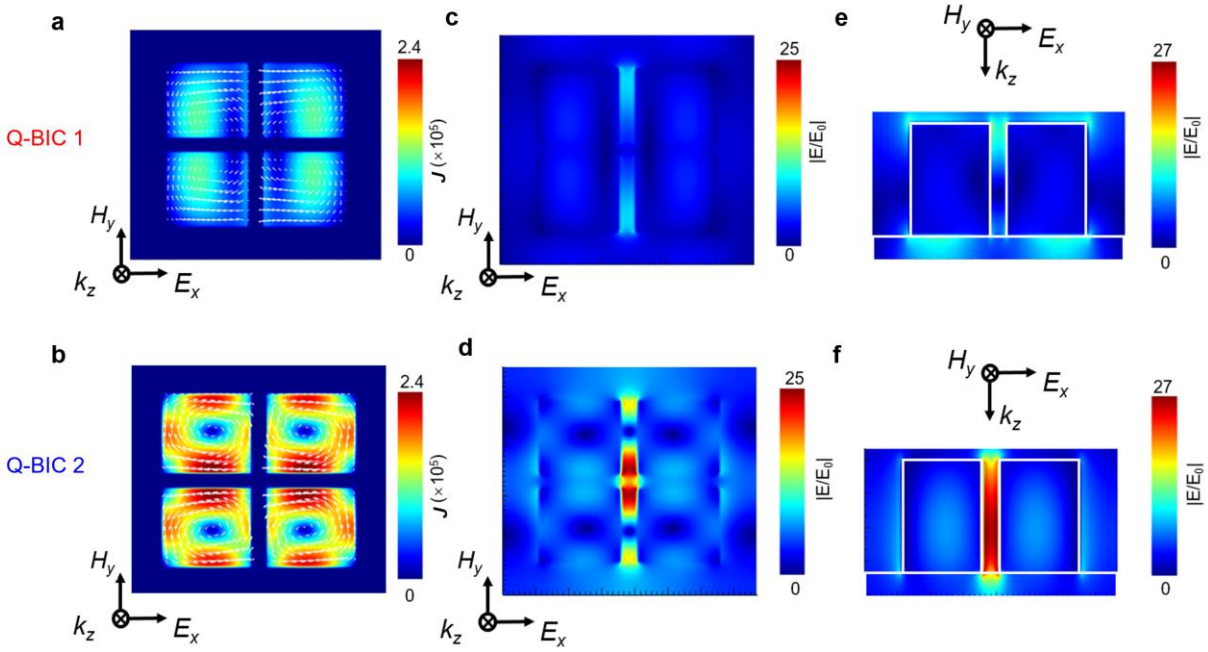

Figure S8 The cross-section patterns of displacement currents (a-b) at the two resonances wavelength of 6050 nm and 6410 nm in the x–y plane for x-polarization. The electric field intensity enhancement $|E/E_0|^2$ at the two resonances in the x–y plane (c-d) and x-z plane (e-f).

Based on the Metaformer model, we develop a software to facilitate the powerful design of metasensor. The software interface is shown in **Figure S9**. We achieve the BRIS and SRIS prediction by the “BRIS” and “SRIS” button after we input the structure parameters. The sensitivity of two models is shown in S1 and S2, respectively. In terms of on-demand deign, we can load the on-demand spectrum to obtain the target structure parameters. We provide three movies to exhibit the software in another supporting information.

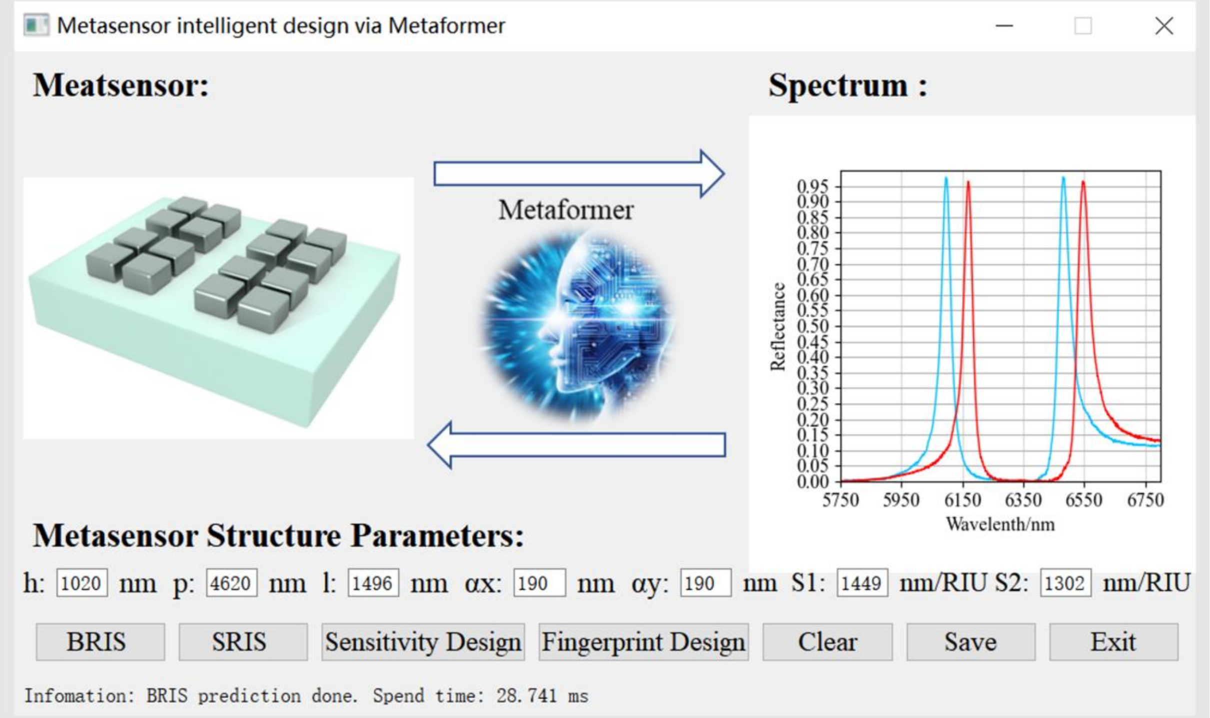

Figure S9 Software interface based on the Metaformer architecture

The fabrication flow based on electron beam lithography is shown in **Figure S10**.

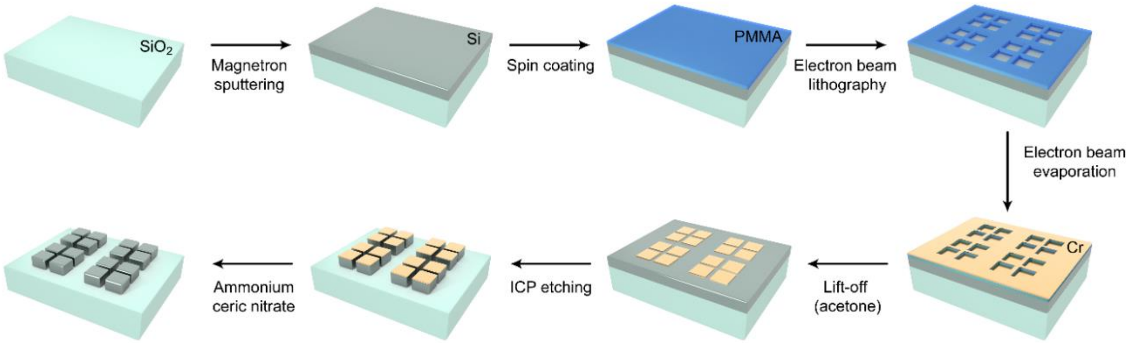

Figure S10 Schematic drawing for the fabrication process of metasensor.

The SEM images of metasensor with Al_2O_3 by a side view is shown in **Figure S11**.

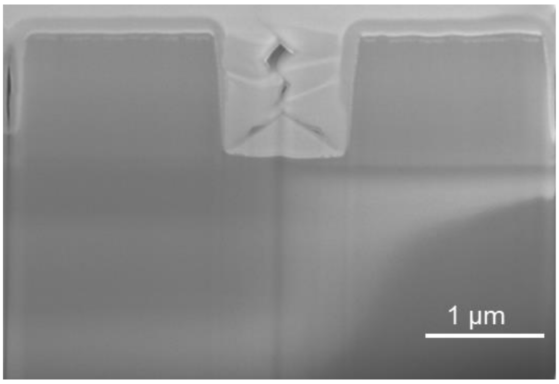

Figure S11 SEM images of Metasensor with Al_2O_3 by side view.

Data Collection for Deep Learning

We randomly collect data by Lumerical FDTD using a desktop (Windows10 operation system, GeForce GTX 3080Ti GPU, Intel(R) Core (TM) i7-10700K CPU @ 3.80GHz 3.79 GHz and 16GB of RAM). The detailed structure parameter range is provided in Table S3.

Table S3 Structure parameters of the metasensor

Structure	Range	Unit
p	4.56 to 4.65	micron
l	1.46 to 1.54	micron
α_x	0.28 to 0.38	micron
α_y	0.28 to 0.38	micron
h	0.98 to 1.02	micron

[1] M. F. Limonov, M. V. Rybin, A. N. Poddubny, Y. S. Kivshar, Fano resonances in photonics. Nature Photonics 11, 543-554 (2017).

[2] Haosen Zhang and Kedi Wu, Ultra-broadband near-perfect absorber based on a single-layer Ge-assisted metasurface, J. Opt. Soc. Am. B.39, 332-337 (2022).

[3] D. P. Kingma, J. L. Ba, Adam: A method for stochastic optimization. International Conference on Learning Representations, (2014).

[4] V. Nair, G. E. Hinton, Rectified linear units improve restricted boltzmann machines. International Conference on Machine Learning, 807-814 (2010).

[5] Deng, H., Zou, N., Du, M., Chen, W., Feng, G., Yang, Z., Li, Z. and Zhang, Q., Unifying fourteen post-hoc attribution methods with taylor interactions. IEEE Transactions on Pattern Analysis and Machine Intelligence. 46, 4625, (2024).

[6] Chen, J., Xu, H., Tao, W., Chen, Z., Zhao, Y., & Han, J. D. J. Transformer for one stop interpretable cell type annotation. Nature Communications, 14(1), 223 (2023).
