# Supplementary figures and images for: Meta‐Attention Deep Learning for Smart Development of Metasurface Sensors
Source: Adv Sci (Weinh). 2024 Sep 9;11(42):2405750. doi: 10.1002/advs.202405750 (PMC11558086; doi:10.1002/advs.202405750)

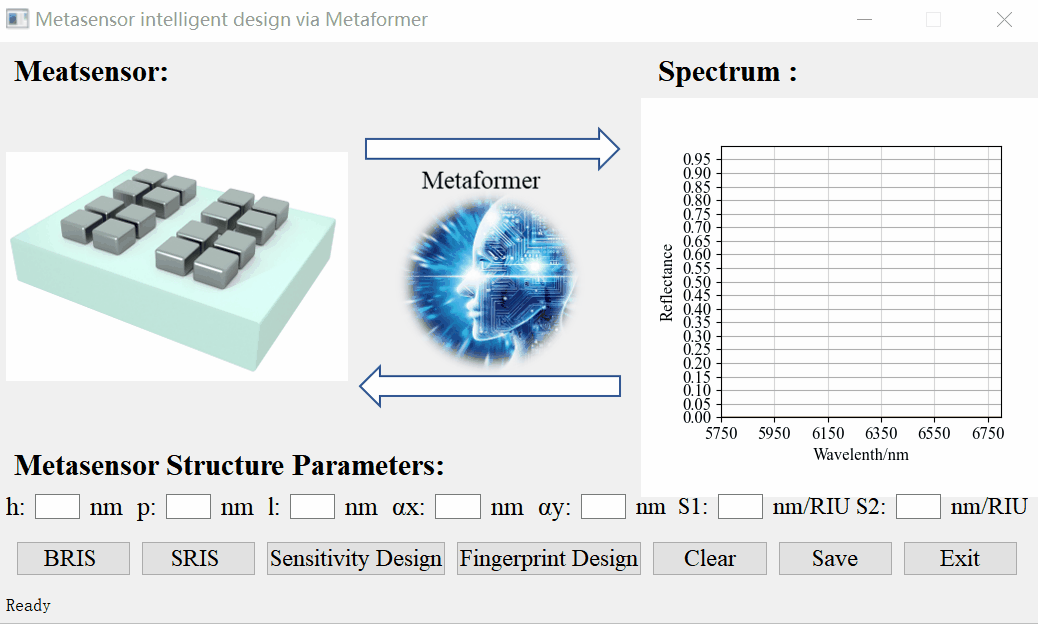

Supplement: Supplementary file 2 — Supporting Information [file ADVS-11-2405750-s001.gif]

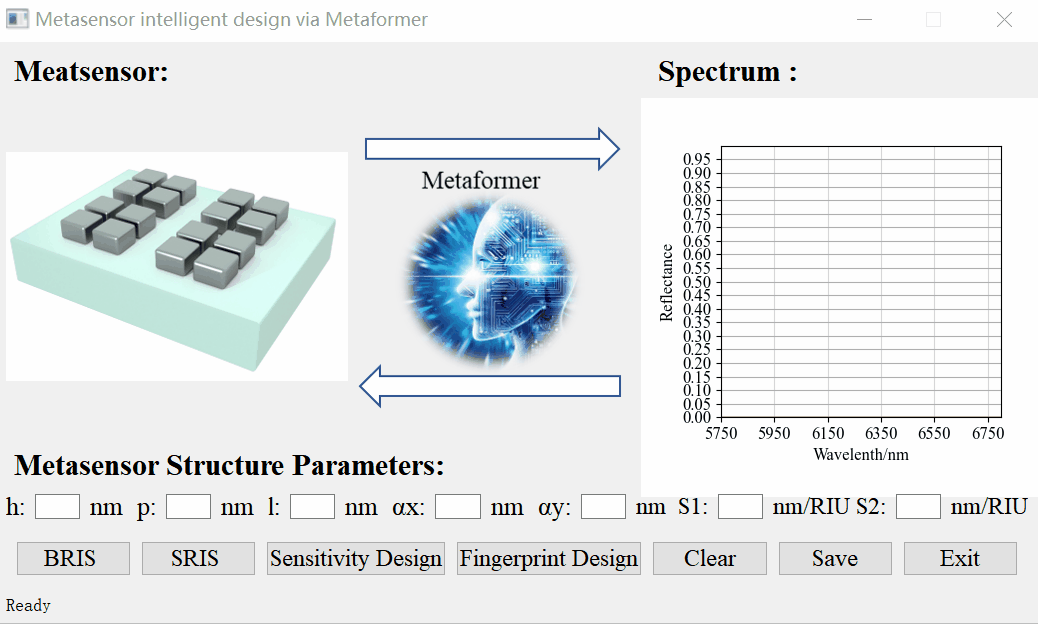

Supplement: Supplementary file 3 — Supporting Information [file ADVS-11-2405750-s004.gif]

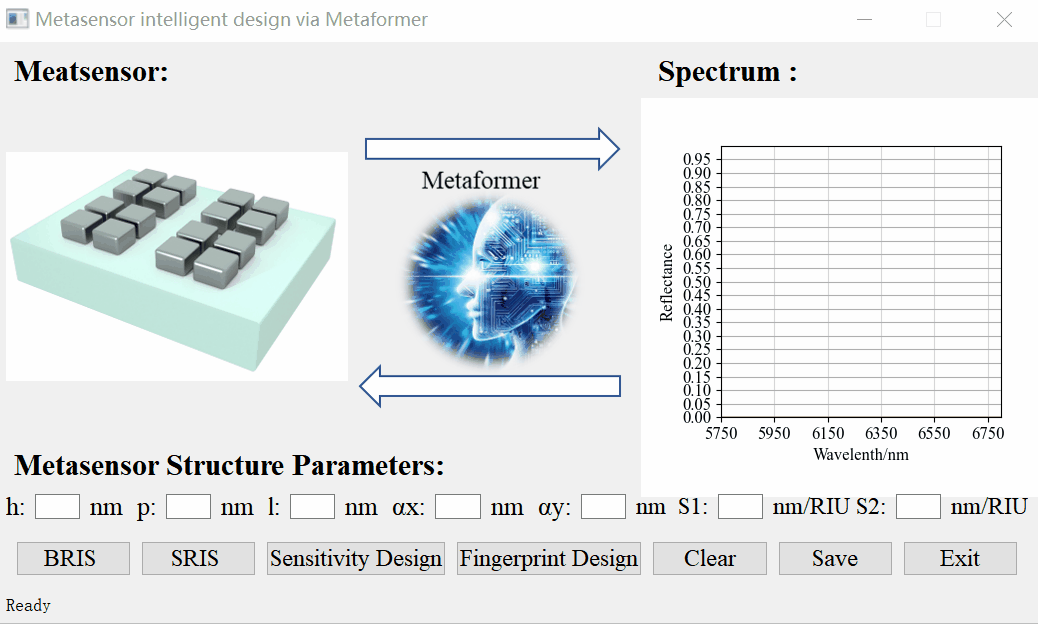

Supplement: Supplementary file 4 — Supporting Information [file ADVS-11-2405750-s003.gif]
